# Supplementary material for: Mesenchymal glioma stem cells trigger vasectasia—distinct neovascularization process stimulated by extracellular vesicles carrying EGFR
Source: Nat Commun. 2024 Apr 3;15:2865. doi: 10.1038/s41467-024-46597-x (PMC10991552; doi:10.1038/s41467-024-46597-x)
Supplement: Supplementary file 1 — Supplementary Information [file 41467_2024_46597_MOESM1_ESM.pdf]

Supplementary figures

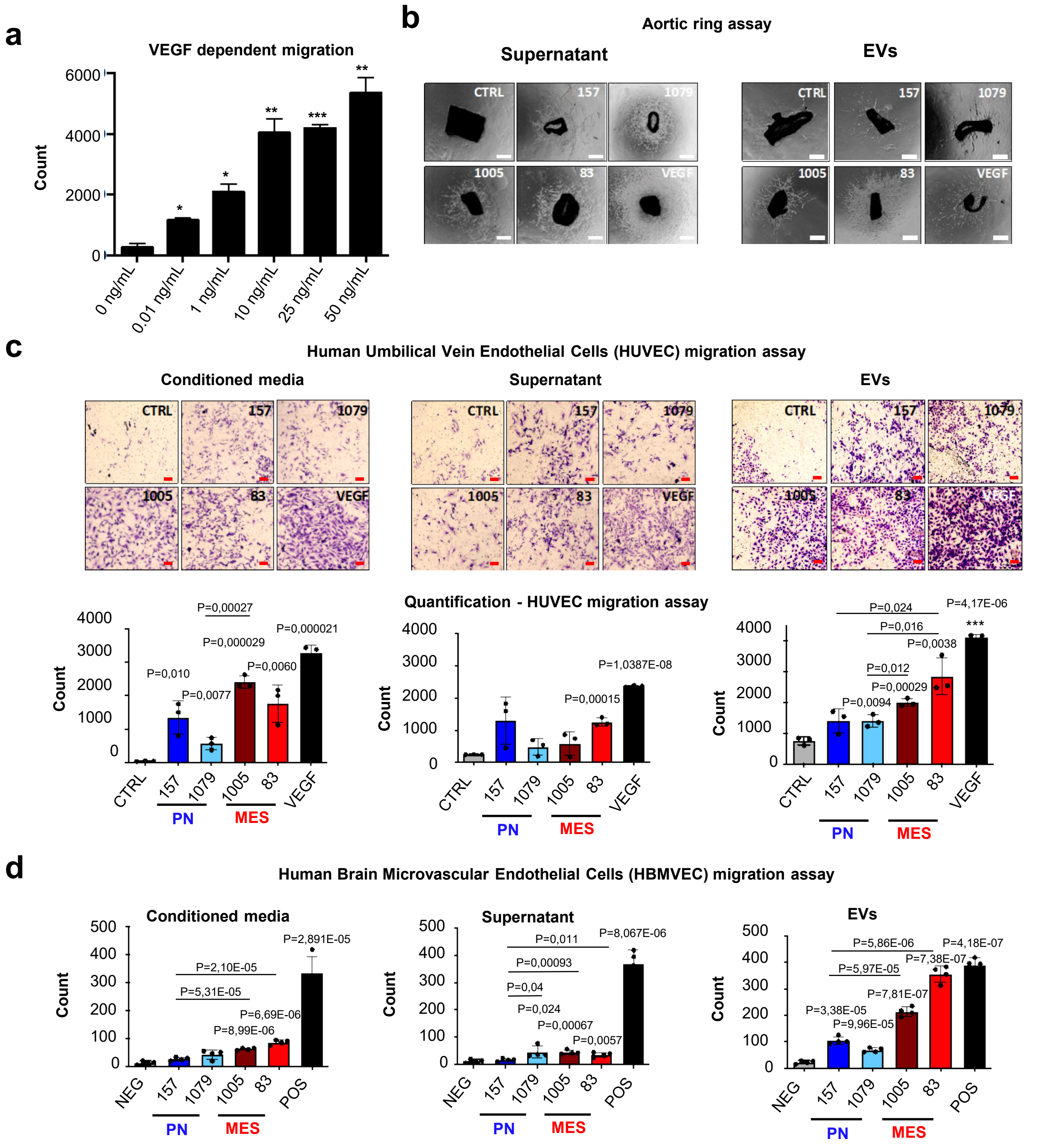

Supplementary Figure 1. Vascular activities of soluble and vesicular components of proneural and mesenchymal glioma stem cell secretomes. a. Concentration-dependent stimulation of endothelial cell (HUVEC) migration by recombinant human VEGF (rhVEGF). After 3 days post stimulation cells were fixed and stained with crystal violet to assess the number of cells migrated through the pores using FIJI software (n=2 independent experiments, two-tailed paired t test; P= 0,012 - 0,013 - 0,0077 - 0,00093 and 0,0053); **b.** Aortic ring assay – endothelial outgrowth responses induced by the whole conditioned media, EV-depleted supernatant and EVs of GSC83 cells (3 days post treatment); **c-d.** HUVEC or HBMVEC transwell migration assay in the presence of whole conditioned media (left), supernatant (middle) or EVs (right) from indicated proneural (PN), or mesenchymal (MES) glioma stem cells (GSCs). Endothelial cells were seeded in growth factors-free media on 0.1% gelatin-coated transwells filters for 24h, then treated as indicated, and 3 days later fixed, stained and enumerated (FIJI software) against rhVEGF positive control (25 ng/mL). Data were presented as means ± SD (n=3 and 4 independent experiments; two-tailed paired t test; * $p < 0.05$, ** $p < 0.01$, *** $p < 0.001$ for treated groups versus controls. Scale bars are 250 µm.

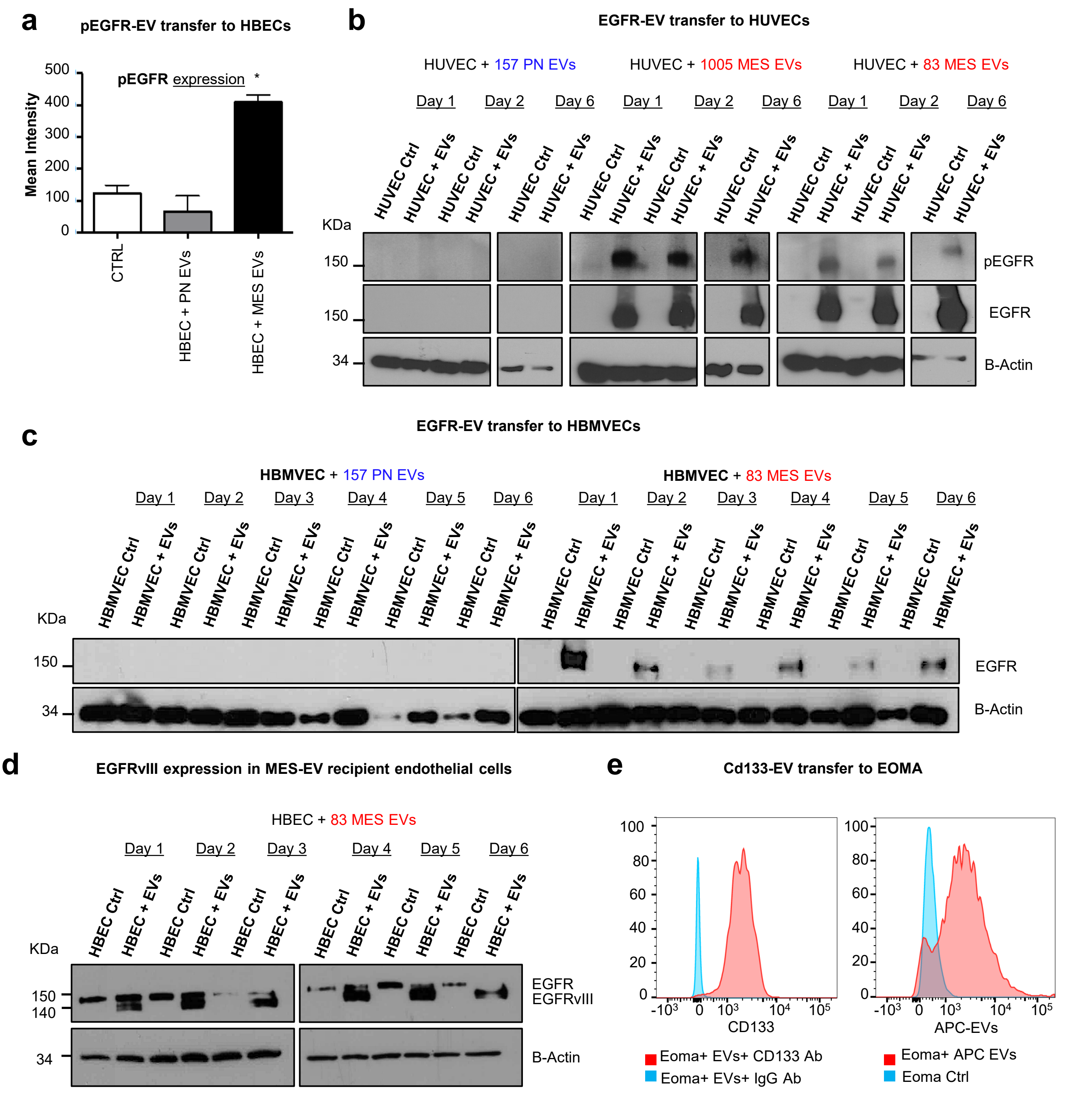

Supplementary Figure 2. Mesenchymal glioma stem cells release extracellular vesicles that transfer to endothelial cells the expression of EGFR/EGFRvIII oncoproteins. **a.** Quantification of the human phospho-RTK signal from the antibody array analysis performed on immortalized human brain endothelial cells (HBEC-5i) treated with EVs from either proneural (PN)- or mesenchymal (MES)-GSC for 7 days. MES-GSC (GSC83) EVs, but not PN-GSC EVs (GSC157) trigger the expression of phosphorylated EGFR in endothelial EV recipients; data presented as means ± SD (n=3 and 4 independent experiments, respectively; two-tailed paired t test; ***$p < 0.001$ for MES-GSC EV-treated group versus no-EV-treated group); **b.** Primary endothelial cells (HUVEC) incubated with EVs from either PN- or MES-GSC EVs were immunoblotted for EGFR, phospho-EGFR (pEGFR) and beta-actin control. Only MES-GSC EVs triggered EGFR expression/phosphorylation in endothelial cells, detectable up to 6 days post treatment (n=3 independent experiments); **c.** EVs from MES (GSC83) but not PN (GSC157) cells transfer the expression of EGFR protein to human brain microvascular endothelial cells (HBMVECs; n=3 independent experiments); **d.** Both EGFR and mutant EGFRvIII are detectable in HBEC-5i endothelial cells following incubation with EVs from GSC83 cells (n=3 independent experiments); **e.** EV uptake and transfer of GSC subtype specific cargoes: CD133 transfer to endothelial cells by EVs from PN GSC donors (Flow cytometry; n=3 independent experiments).

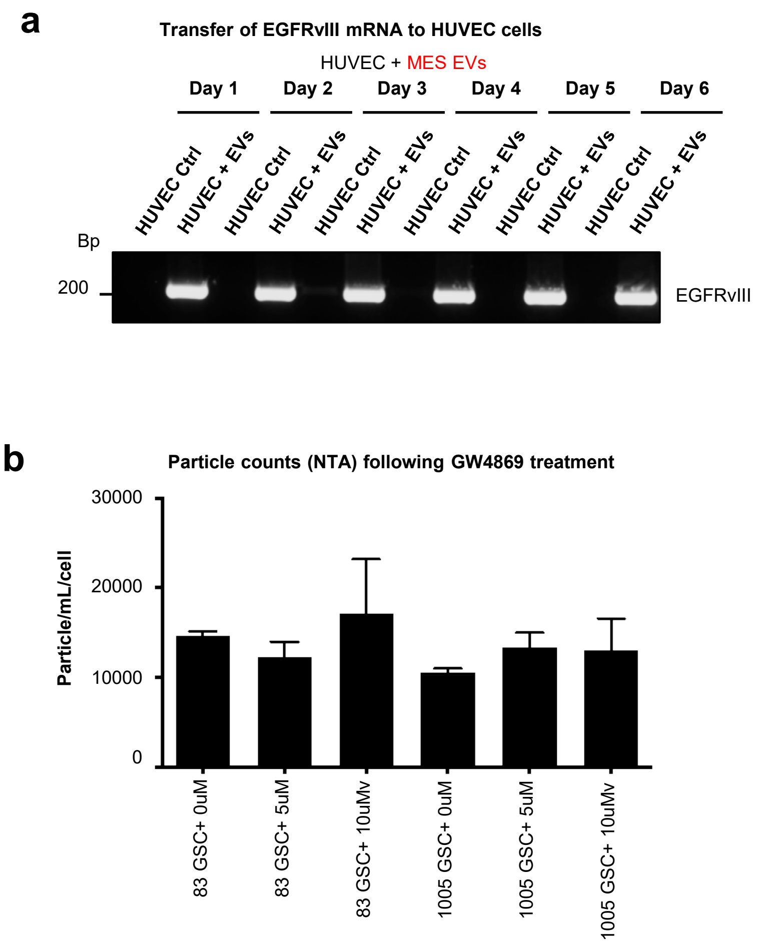

Supplementary Figure 3. Characterization of glioma stem cell derived extracellular vesicles. a. Mesenchymal glioma stem cell (GSC83)-derived EVs transfer EGFRvIII mRNA to human primary endothelial cells (HUVEC; signal detected up to 6 days post treatment; n=3 independent experiments); **b**. Nanoparticle tracking (NTA) analysis of EVs released into cell culture media following treatment of MES-GSCs (GSC83, GSC1005) with different concentrations of GW4869 inhibitor (5 -10 μM) for 24h; the levels of vesiculation remained relatively unchanged. (n=3 independent experiments).

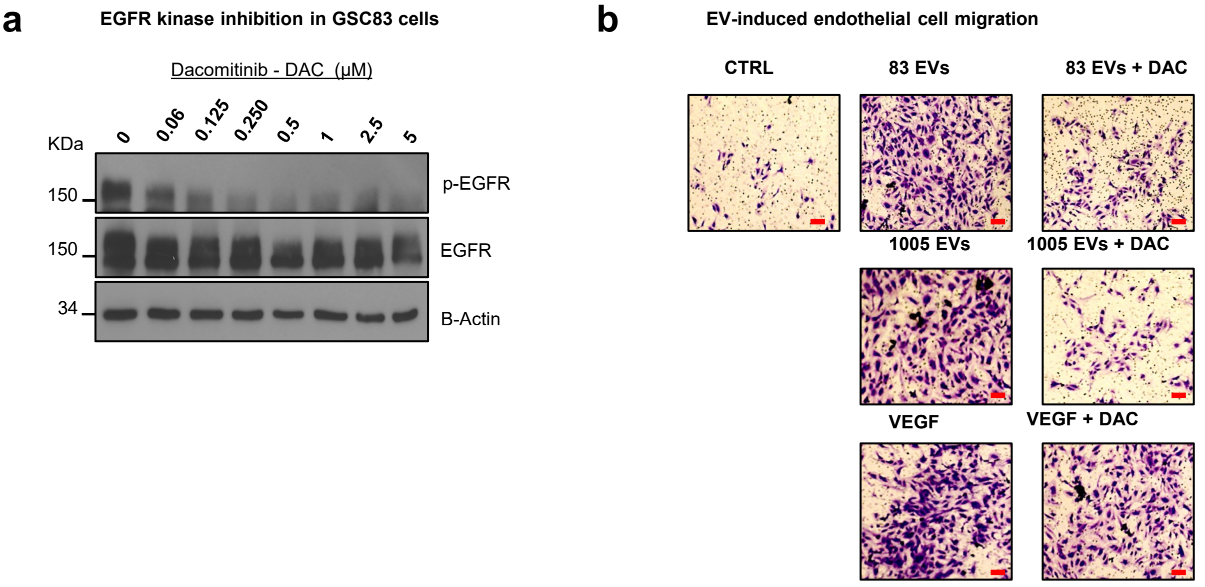

Supplementary Figure 4. Pharmacological targeting of EGFR in EV donor cells. a. Effects of different Dacomitinib (DAC) concentrations on EGFR phosphorylation in mesenchymal glioma stem cells. GSC83 cultures were treated with various concentrations of DAC (0.06 µM to 5 µM) for 24h after which the cells were analyzed by western blot. DAC exposure led to near complete loss of EGFR phosphorylation at 0.250 µM and above. (n=3 independent experiments). **b.** DAC inhibition of endothelial cell migration stimulated by EGFR-carrying EVs. Endothelial cells were seeded in growth factors-free media on 0.1% gelatin-coated transwells. After 24h they were treated with MES EVs, or VEGF and 6 hours later with 2.5 µM of Dacomitinib. After a period of 3 days, cells were fixed and stained with crystal violet to assess the number of cells migrated (n=3 independent experiments).

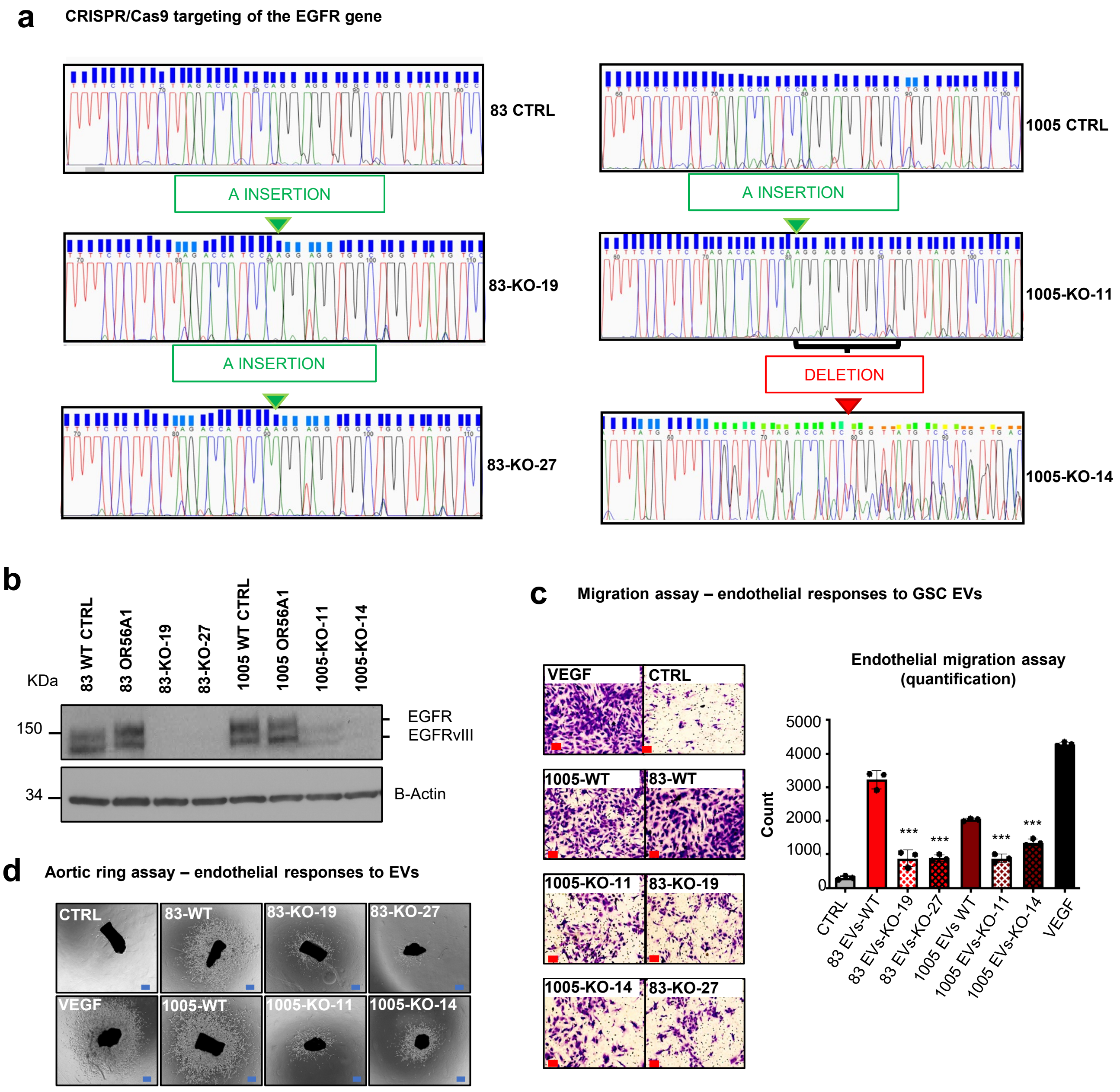

Supplementary Figure 5. Genetic targeting of EGFR in EV donor cells. a. Sanger electropherograms of short PCR products around the gRNA regions in Cas9-gRNA transduced single clones of indicated MSE-GSC lines. Genetic analysis of EGFR indicates the insertion of a single adenine for 83-KO-19, 83-KO-27 and 1005-KO-11, and for MES-GSC1005-KO-14 a 10-nucleotide deletion. All the CRISPR/Cas9-mediated genome editing induced a disruption in gene expression and function; **b.** Validation of EGFR knockout by western blotting. (n=3 independent experiments); **c.** EGFR depletion reduced the ability of GSC EVs to trigger endothelial cell migration. Endothelial cells were treated with 30 µg/mL of EVs derived from cells deficient (EGFR-KO) or proficient (EGFR-WT) for EGFR. After 3 days cells were fixed, stained with crystal violet and imaged. (n=3 independent experiments; two-tailed paired t test; Significance of difference for GSC83-EVs: P=0,00036 and 0,00014; for GSC1005-EVs: P=9,84E-05 and 0,00037); **d.** EGFR depletion reduced the ability of EVs to trigger endothelial cell outgrowths from aortic rings. Isolated rings were treated with 30 µg/mL of EVs obtained from GSCs with EGFR-KO or EGFR-WT status. For both assays 25 ng/mL of rhVEGF was used as a positive control (n=3 independent experiments; data were presented as means ± SD; **p < 0.01, ***p < 0.001 for treated group versus controls; Scale bar in red is 250 µm; Scale bar in blue is 0.5 mm).

a

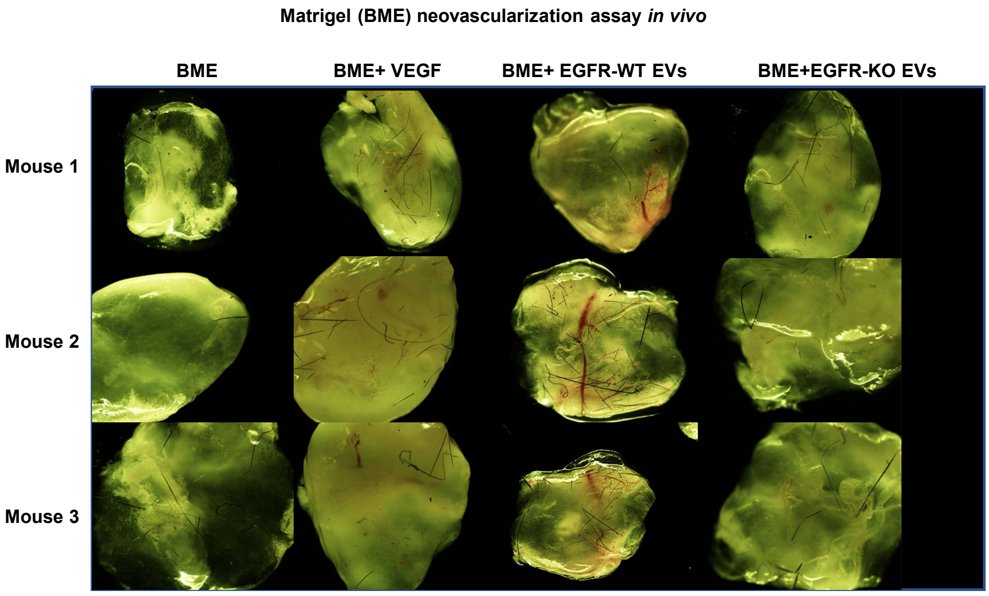

Supplementary Figure 6. BME plug assay for assessment of blood vessel stimulating activities of tumour cell derived extracellular vesicles in the absence of cancer cells. EVs were isolated from cultures of EGFR-proficient (WT) and -deficient (KO) glioma stem cells (GSC83) and added to BME matrix before subcutaneous injection into immune deficient NSG mice. Recombinant VEGF (25 ng/mL) was addad, as a positive control. Ten days post injection BME plugs were removed and photographed (n=3 independent experiments).

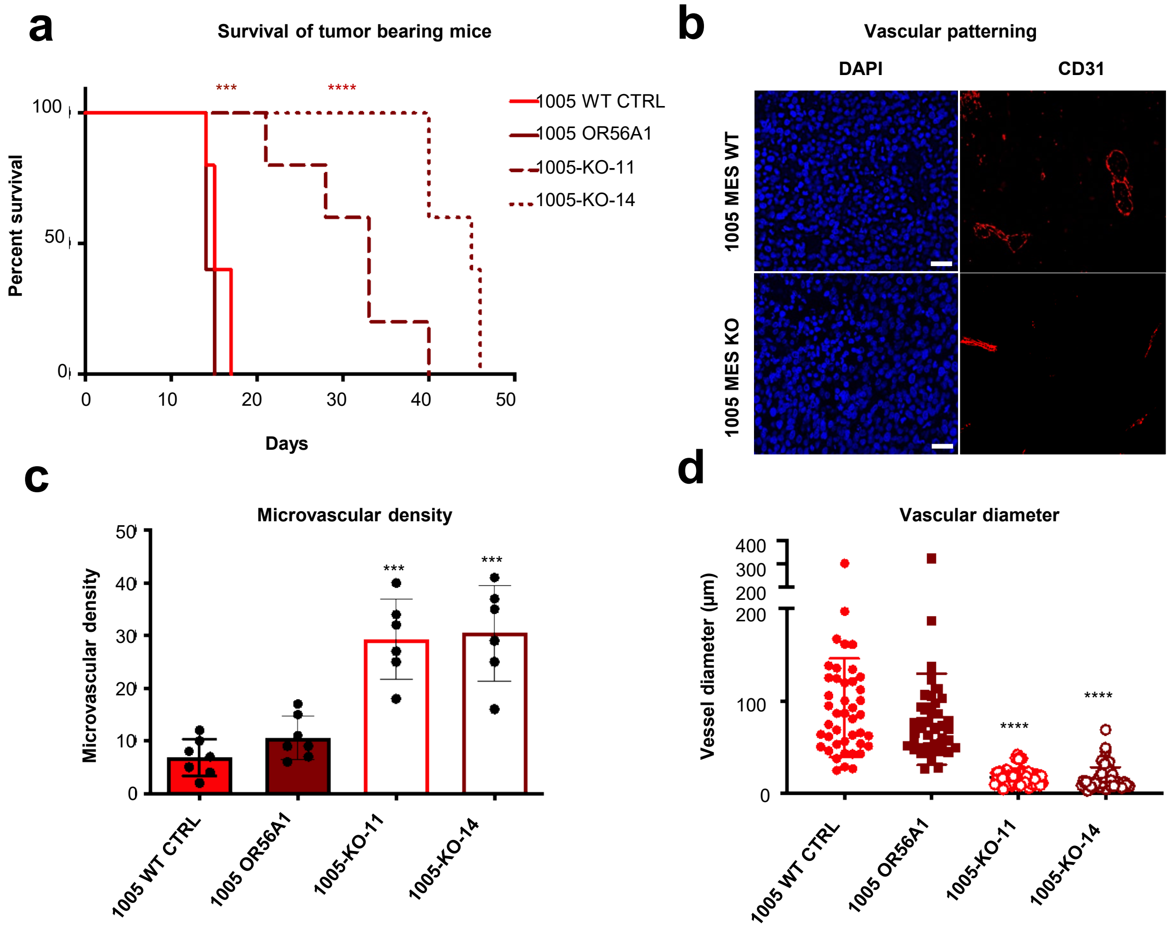

Supplementary Figure 7. Disruption of *EGFR* gene in mesenchymal glioma stem cells results in diminished tumour aggressiveness and abrogation of vasectasia-like vascular patterns. a. Impact of *EGFR* gene editing on GSC1005 xenografts; intracranial tumour growth in mice was measured by symptom free survival – comparison of two independent EGFR-KO clones (1005-KO-11 and 1005-KO-14) and two controls (1005 WT and 1005 OR56A1; n=5 independent experiments; two-tailed paired t test; P= 0.000103 and 0.000010); **b** Representative images of immunofluorescence for CD31 reveals differential vascular patterns between tumours driven by MES GSCs with different EGFR status: EGFR-WT or MES GSC EGFR-KO; n=5 independent experiments); **c.** Quantification of vessel size distribution through CD31 staining of endothelial cells (n=5 independent experiments; two-tailed paired t test; P= 2,32^{-05} and 5,23^{-05}); **d.** Quantification of microvascular density using CD31 staining (n=5 independent experiments; two-tailed paired t test; P= 7,40^{-16} and1,30^{-16}). Microvascular density was expressed as vessel numbers per high power field (hpf; scale bars are 50 µm).

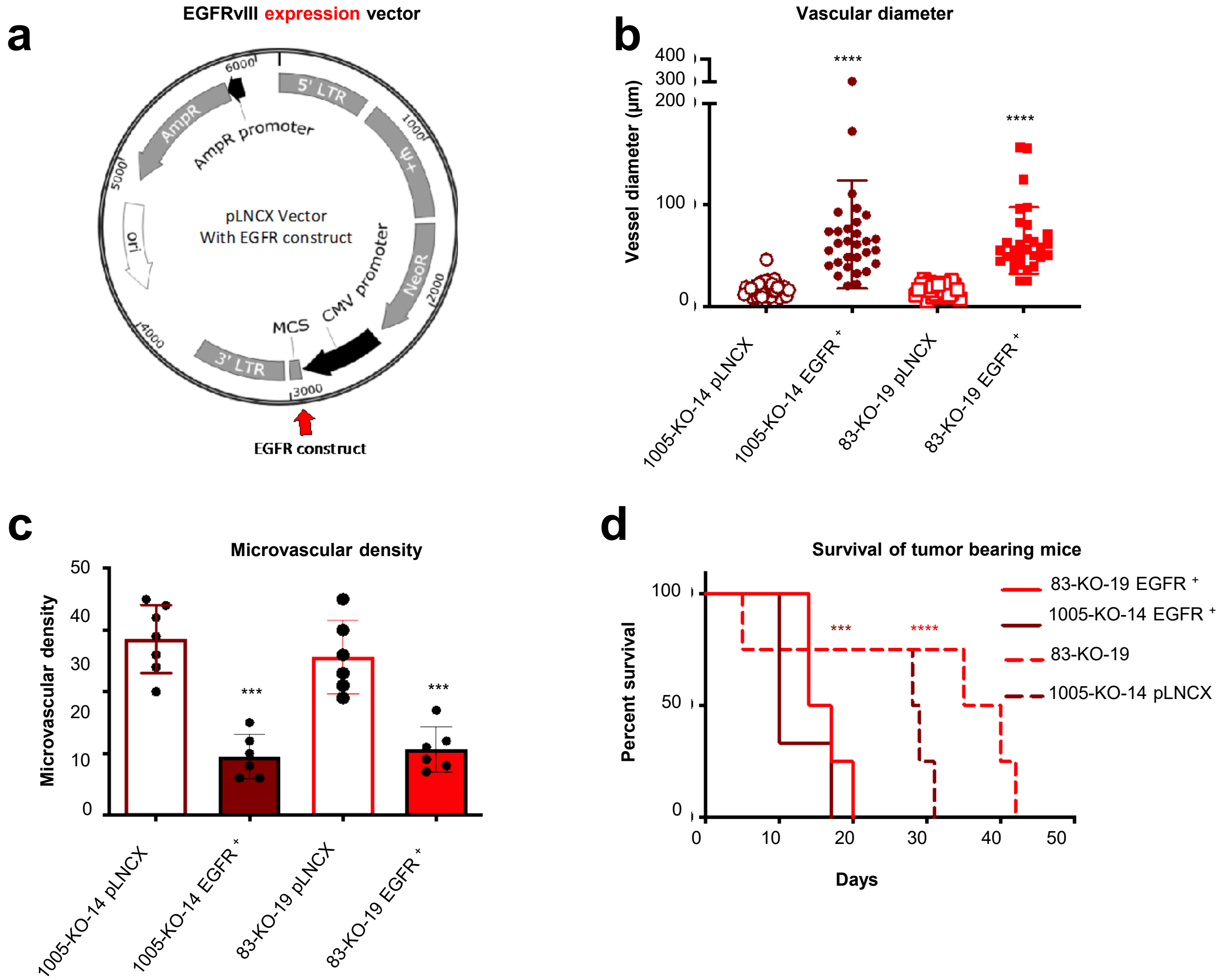

Supplementary Figure 8. Rescue of the agressive phenotype and vasectesia-like vascular patterns upon selective re-expression of EGFRvIII in mesenchymal GSCs with disrupted *EGFR* gene. a. EGFRvIII expression vector; vascular properties of GSC83 and GSC1005 intracranial xenografts with disrupted EGFR, and either untransfected (83-KO-19), transfected with empty vector (1005-KO-14-pgLNCX), or reconstituted for selective EGFRvIII expression (83-KO-19-EGFR+; 1005-KO-14-EGFR+); **b.** Quantification of vessel size distribution through CD31 staining of endothelial cells (n=5 independent experiments; two-tailed paired t test P= $1{,}19^{-06}$ and $7{,}67^{-11}$); **c.** Quantification of microvascular density using CD31 staining (n=5 independent experiments; two-tailed paired t test; P= $1{,}68^{-05}$ and 0,00037); Microvascular density was expressed as vessel numbers per high power field (hpf). **d.** Kaplan-Meier survival curves of mice bearing EGFR-WT or EGFR-KO MES GSC-derived tumors.

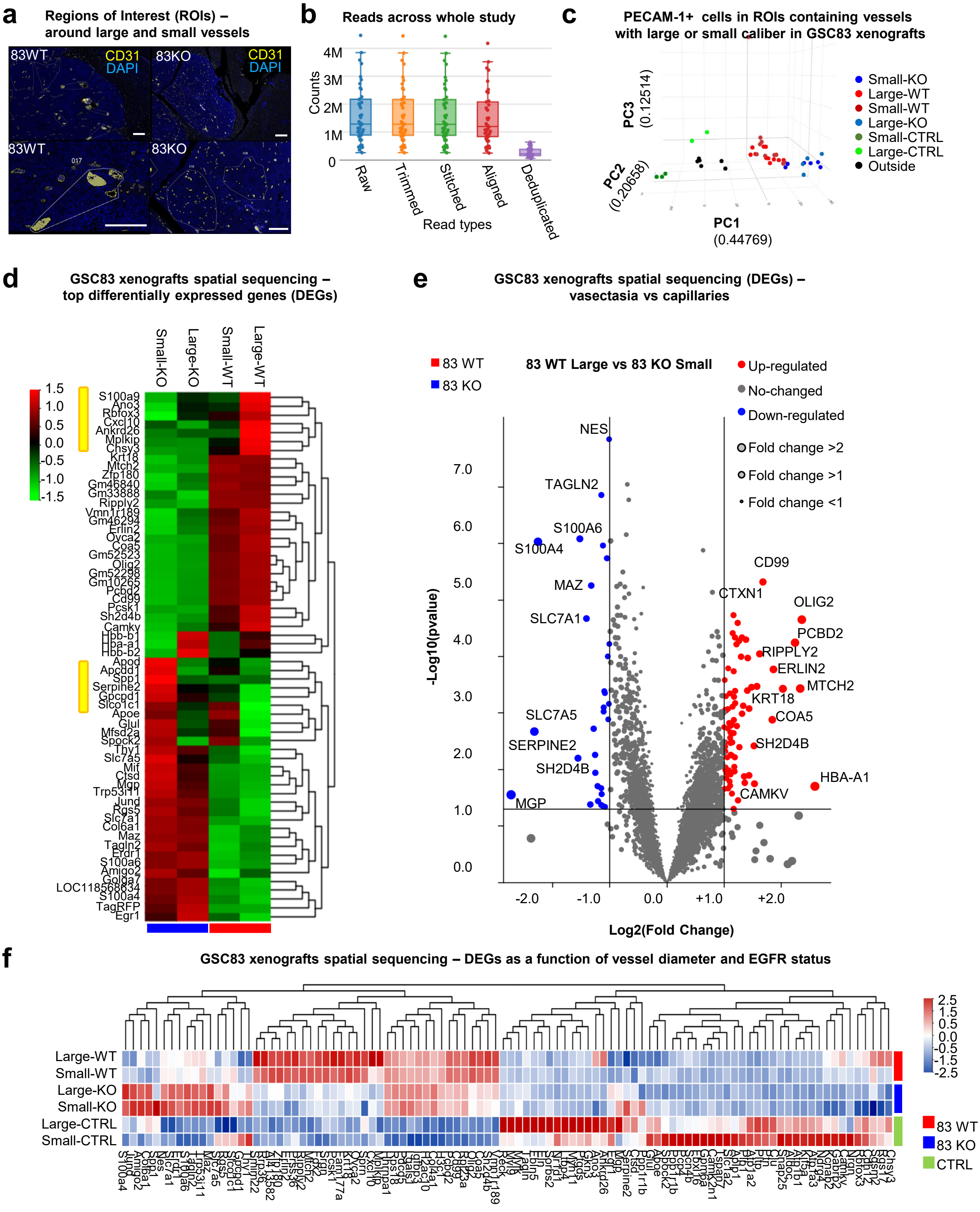

Supplementary Figure 9. Spatial gene expression profiling (GeoMX) in GSC83 intracranial tumour regions containing large (extended) and small (capillary) vascular structures. **a.** Regions of interest (ROIs) around vessels highlighted with anti-CD31 fluorescent immunostaining; **b.** Characteristics of sequencing reads; **c.** Clustering of transcriptomes in ROIs; **d.** Differentially expressed genes (DEGs) in indicated ROIs containing large or small vessels, as a function of either wild type (WT) or knock-out (KO) EGFR status; **e.** Volcano plot of statistical differences in DEG between ROIs containing capillary (small - KO tumours) and vasectasia-type (large - WT tumours) vasculatures; **f.** Comparison between trascriptomes in ROIs containing small or large vascular structures in WT or KO tumours, versus corresponding vessel calibers in control mouse brains.

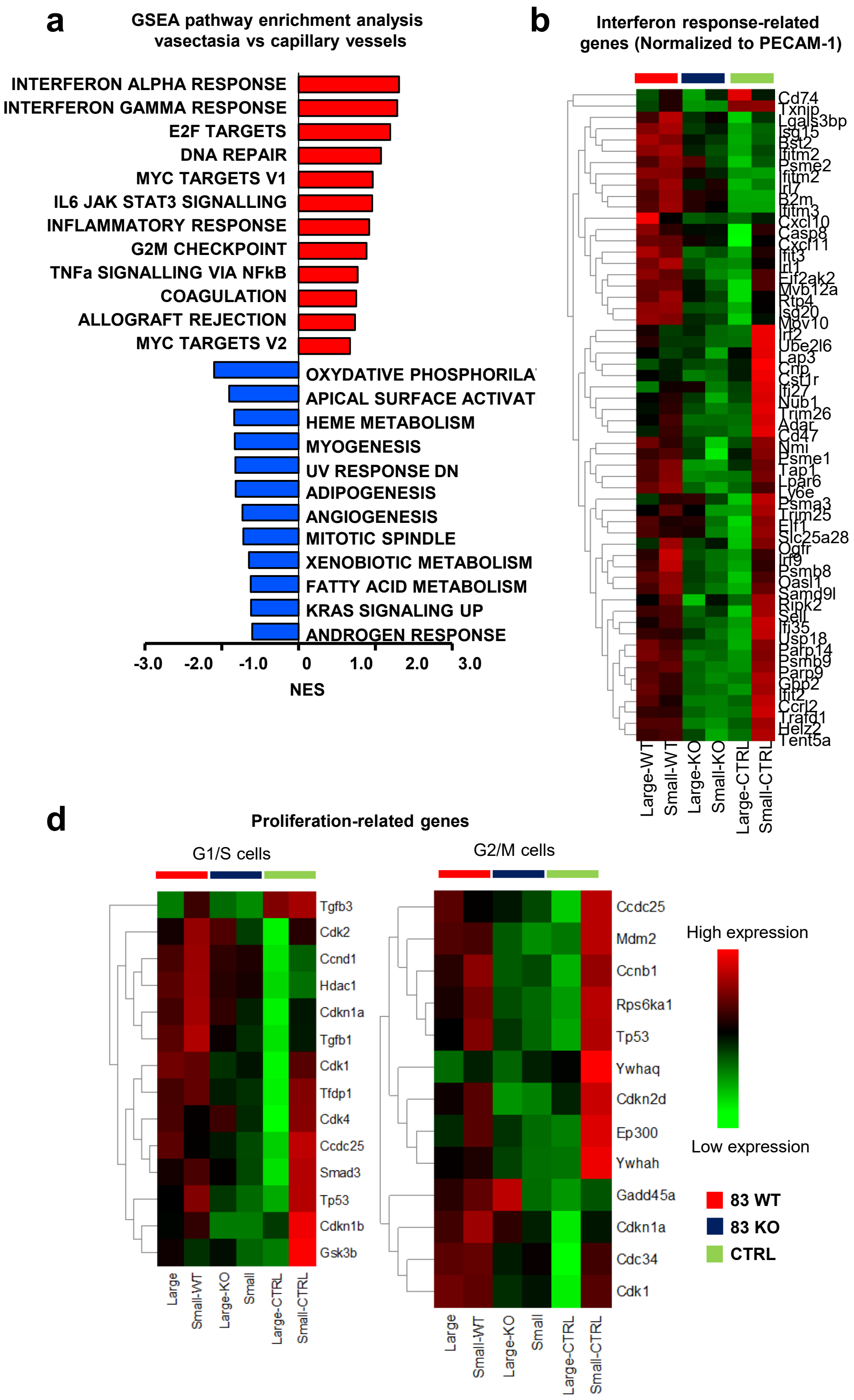

Supplementary Figure 10. Spatial expression profiling (GeoMX) of gene subsets in vascular regions of brain tumours driven by GSCs that are either proficient or deficient for EGFR/EGFRvIII. a. GSEA analysis of pathways enriched in vascular regions (ROIs) containing large vessels (83 WT tumours – EGFR/EGFRvIII expressors), or small/capillary vessels (83 KO tumours – EGFR/EGFRvIII non-expressors) normalized to normal brain vessels; NES = normalized enrichment score); **b**. Differentially expressed genes (DEGs) related to interferon response within indicated ROI; **c.** DEGs related to angiogenesis within indicated ROI; **d.** DEGs related to cellular proliferation including expression associated with G1/S or G2/M phases of cell cycle.

a

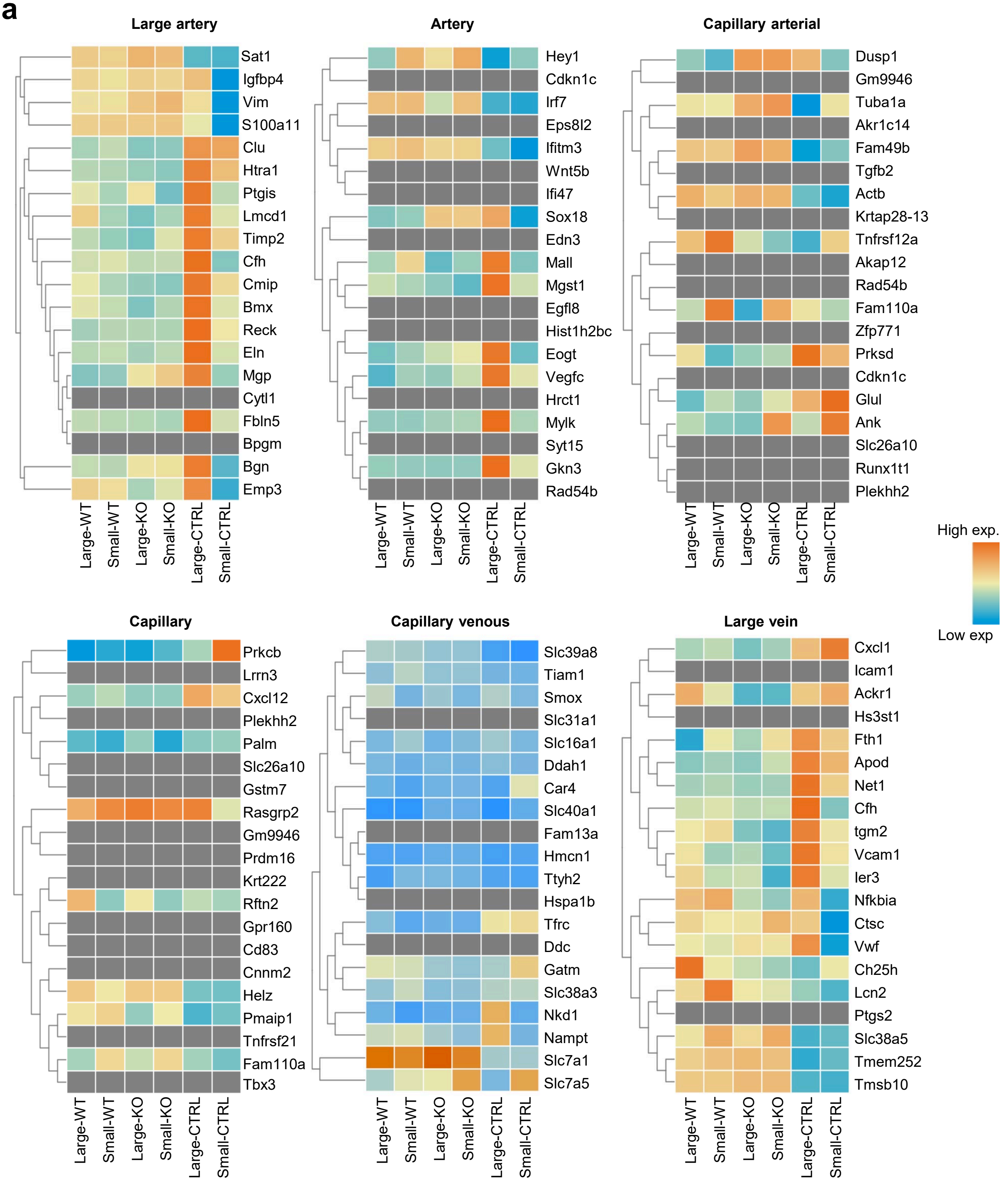

Supplementary Figure 11. Divergence of GeoMx mRNA profiles between glioma xenograft-associated vascular regions and regions related to vascular structures of corresponding sizes in normal mouse brain. Transcriptomes of endothelial cells in key categories of normal blood vessels extracted from the ATLAS of normal mouse brain vasculature (*Vanlandewijck et al 2018*) were compared to the expression levels of the respective genes in vascular ROIs containing large or small vessels in EGFR-proficient (83 WT) or –deficient (83 KO) intracranial xenografts.

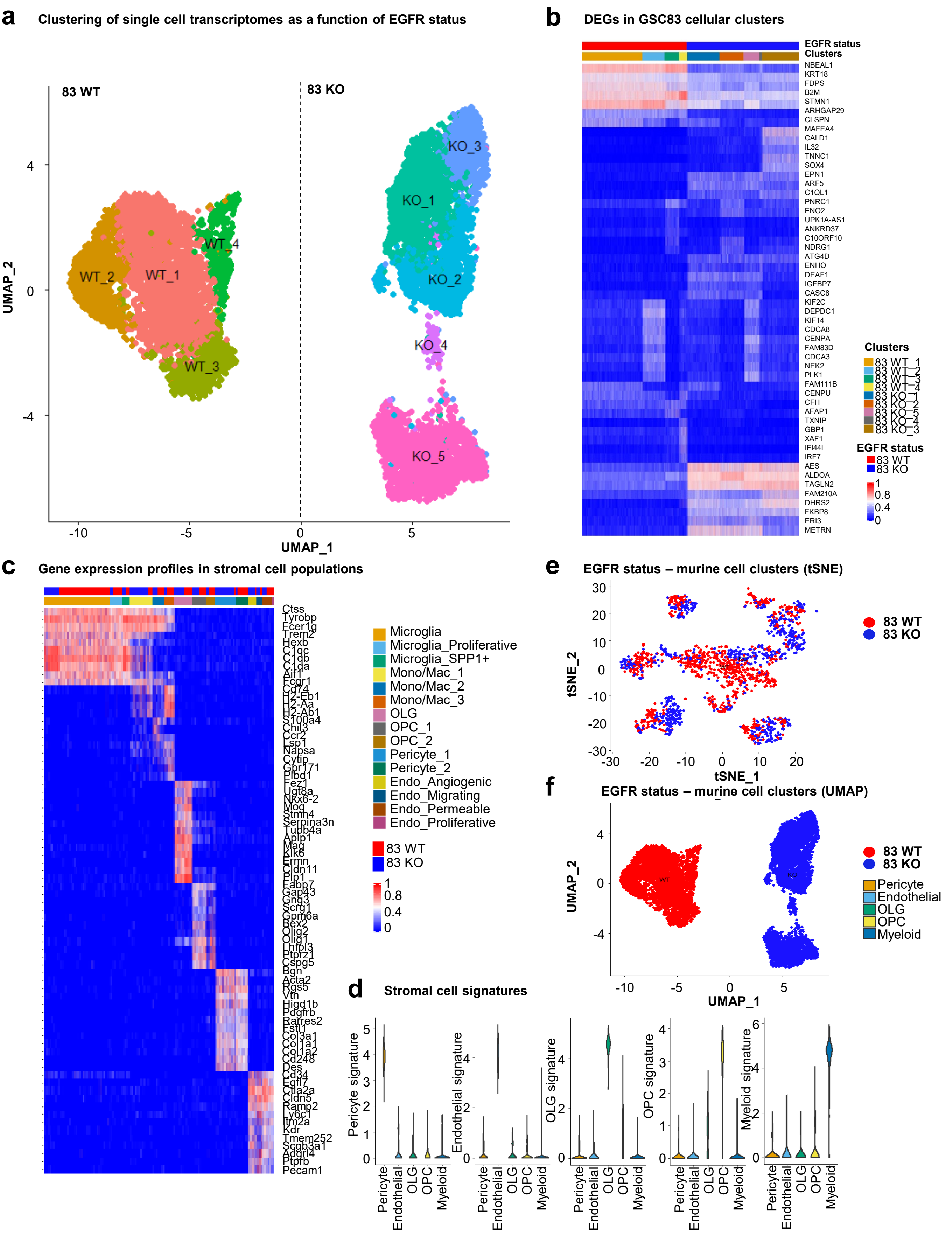

Supplementary Figure 12. Distinctive cellular landscapes of EGFR/EGFRvIII-proficient and –deficient brain tumours. Single cell RNA sequencing (scRNAseq) analysis of cell populations in intracranial xenografts driven by EGFR-expressing (GSC83-WT) and EGFR-disrupted (GSC83-KO) mesenchymal glioma stem cells. **a.** Distinct clusters of cells in EGFR-WT and EGFR-KO tumours; **b.** Differentially expressed genes (DEGs) in indicated tumours and cellular clusters; **c.** Gene expression levels of top-ranking marker genes in different murine populations identified in GSC EGFR-WT and EGFR-KO tumours; **d.** Transcriptional signatures of vascular and stromal cells in brain tumours; **e.-f.** tSNE plot of murine cells populations and UMAP plot of human glioma stem cells transcriptomes color coded by EGFR status.

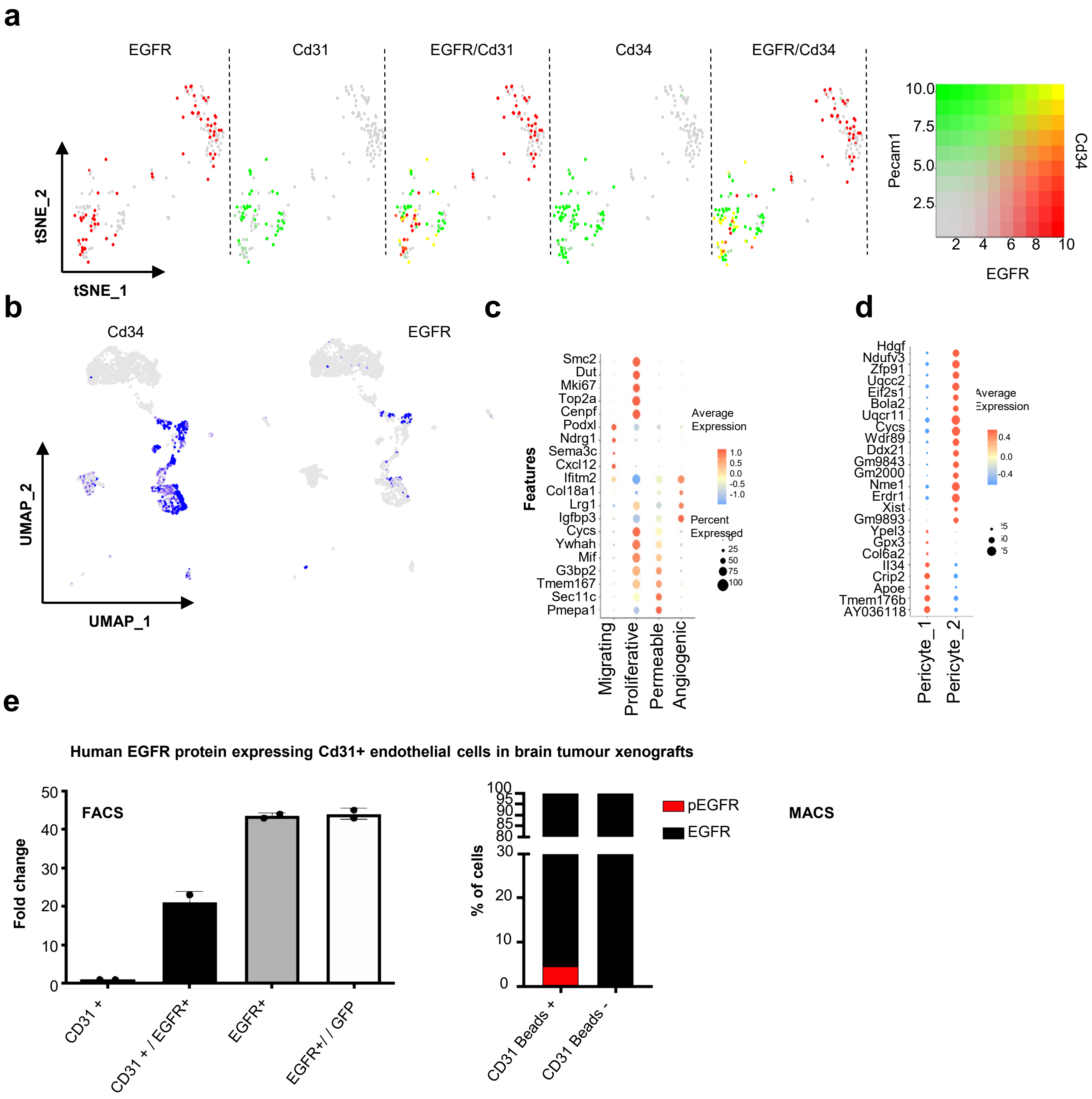

Supplementary Figure 13. Gene expression profiles of stromal/murine cells in brain xenografts, as a function of EGFR/EGFRvIII status of cancer cells. a. Subsets of murine endothelial cells expressing human EGFR as determined by Cd31 and Cd34 co-expression (GSC83-EGFR-KO cells express dysfunctional but detectable EGFR transcript). **b** t-SNE plots of human glioma cells transcriptomes identify CD34 and EGFR colocalization in patient samples; **c.** Dot plot showing differential expression patterns of marker genes in individual clusters of endothelial cells. **d.** Dot plot showing differential expression patterns of marker genes in individual clusters of pericyte cell; **e.** Validation of human EGFR+ mouse endothelial cells in GSC83 tumours by cell sorting (FACS) and EGFR phosphorylation by magnetic bead separation (MACS; n=2 independent experiments).

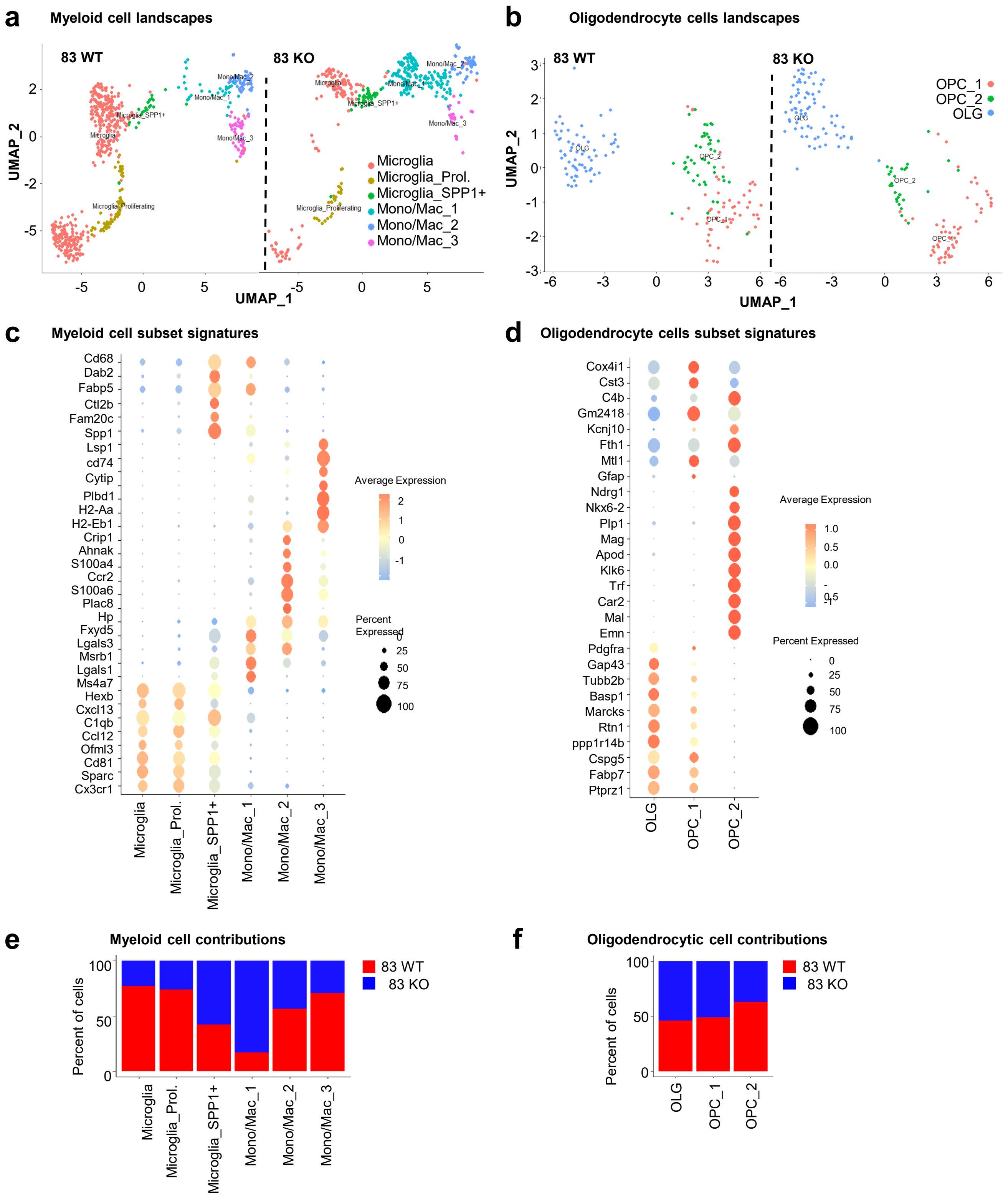

Supplementary Figure 14. Impact of tumour EGFR status on myeloid and oligodendrocytic stromal cells in intracranial glioma xenografts in mice. a. tSNE plot of murine cells shows clustering based on gene expression. Three different populations of microglia and myeloid are detected; **b.** tSNE plot reveals different populations oligodendrocyte and their precursors. **c.** Dot plot showing differential expression patterns of marker genes in individual clusters of microglia and myeloid cells; **d.** Dot plot showing differential expression patterns of marker genes in individual clusters of oligodendrocyte and precursor cells; **e.** Relative proportion of oligodendrocyte and their precursor cells populations in either EGFR-KO or EGFR-WT tumours; **f.** Relative proportion of oligodendrocyte and precursor cells subtypes in either EGFR-KO or EGFR-WT tumours.

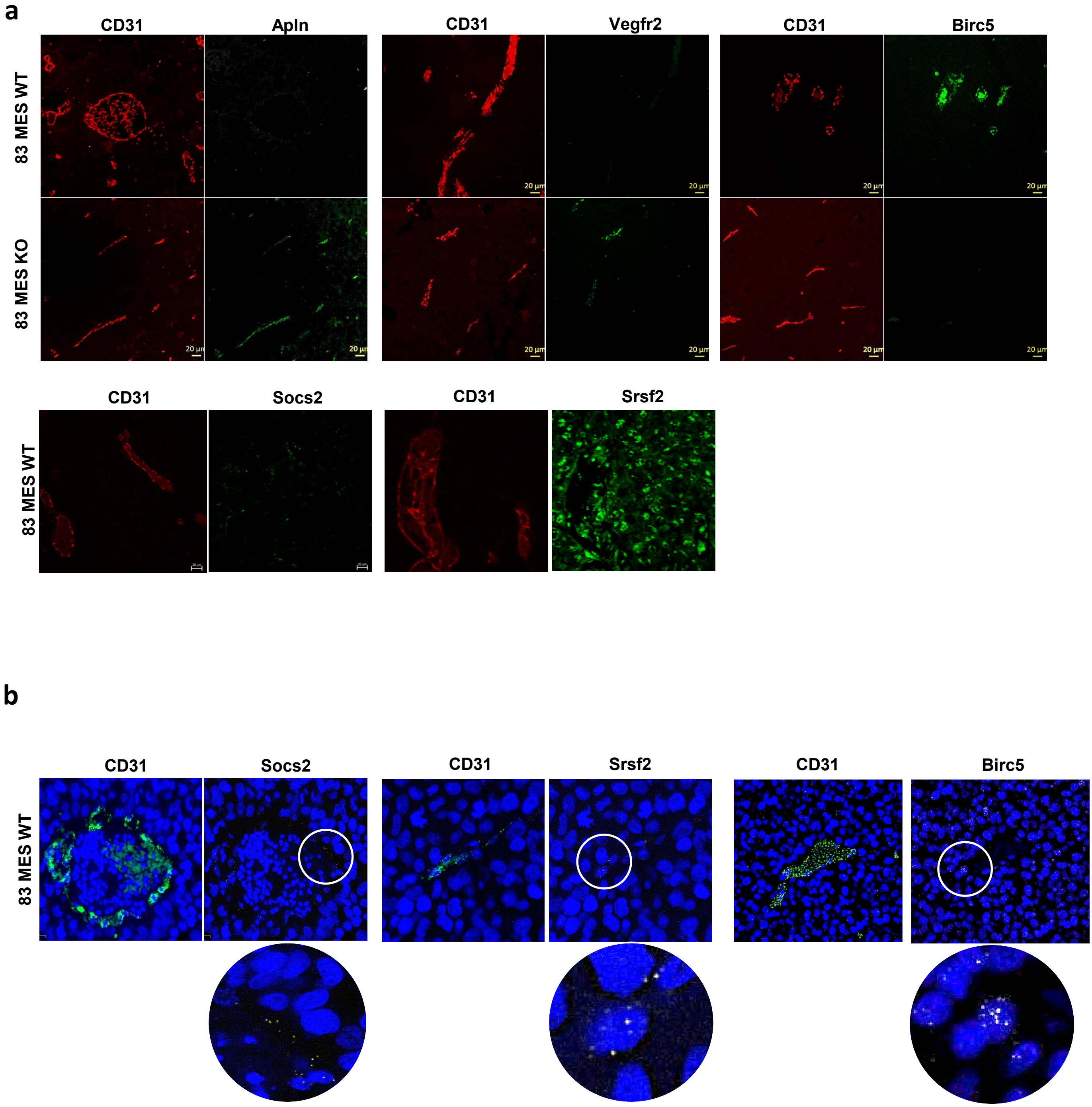

Supplementary Figure 15. Expression of mRNA and protein markers detected in CD31 positive endothelial cells in mesenchymal glioma stem cell xenografts in mice. **a.** Representative images of immunofluorescence for CD31 combined with selected markers: Apln, VEGFR2, Birc5, Socs2 and Srsf2 reveals molecular differences associated with vascular patterns (N=3); **b.** *In-situ* hybridization (ISH) assay, using RNAscope- spatial gene expression platform, for detection of target RNAs within intact cells. Socs2, Srsf2 and Birc5 mouse specific transcripts were validated in mouse xenograft tissues.

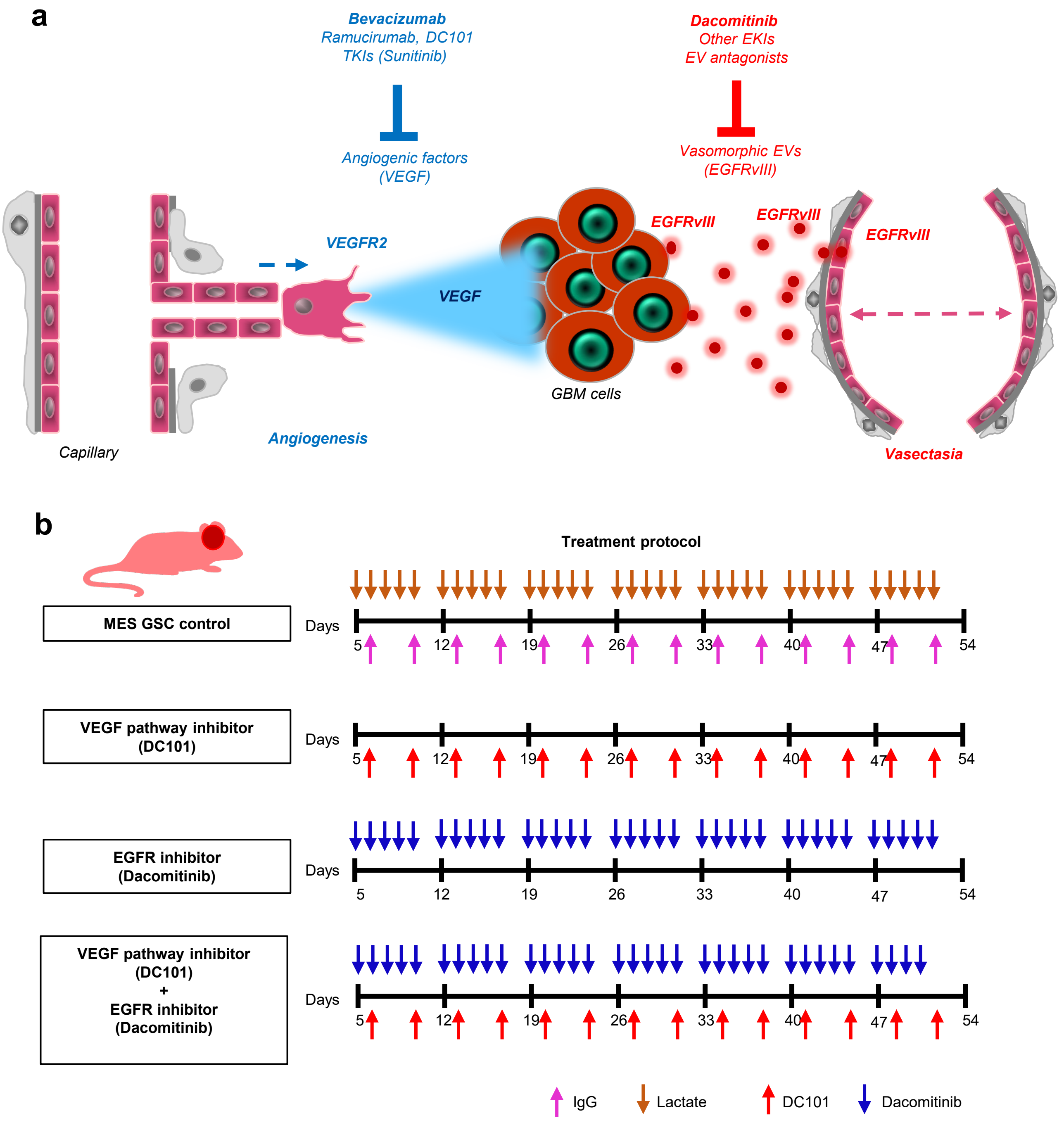

Supplementary Figure 16. Strategies of blood vessel targeting in glioblastoma. a. Model of glioblastoma neovascularization through combined effect of angiogenesis and vesectasia – therapeutic implications; **b.** Treatment protocol targeting VEGF pathway of angiogenesis (DC101) and EGFR pathway of vasectasia (Dacomitinib) in human mesenchymal glioma stem cell xenografts in immune deficient (NSG) mice.

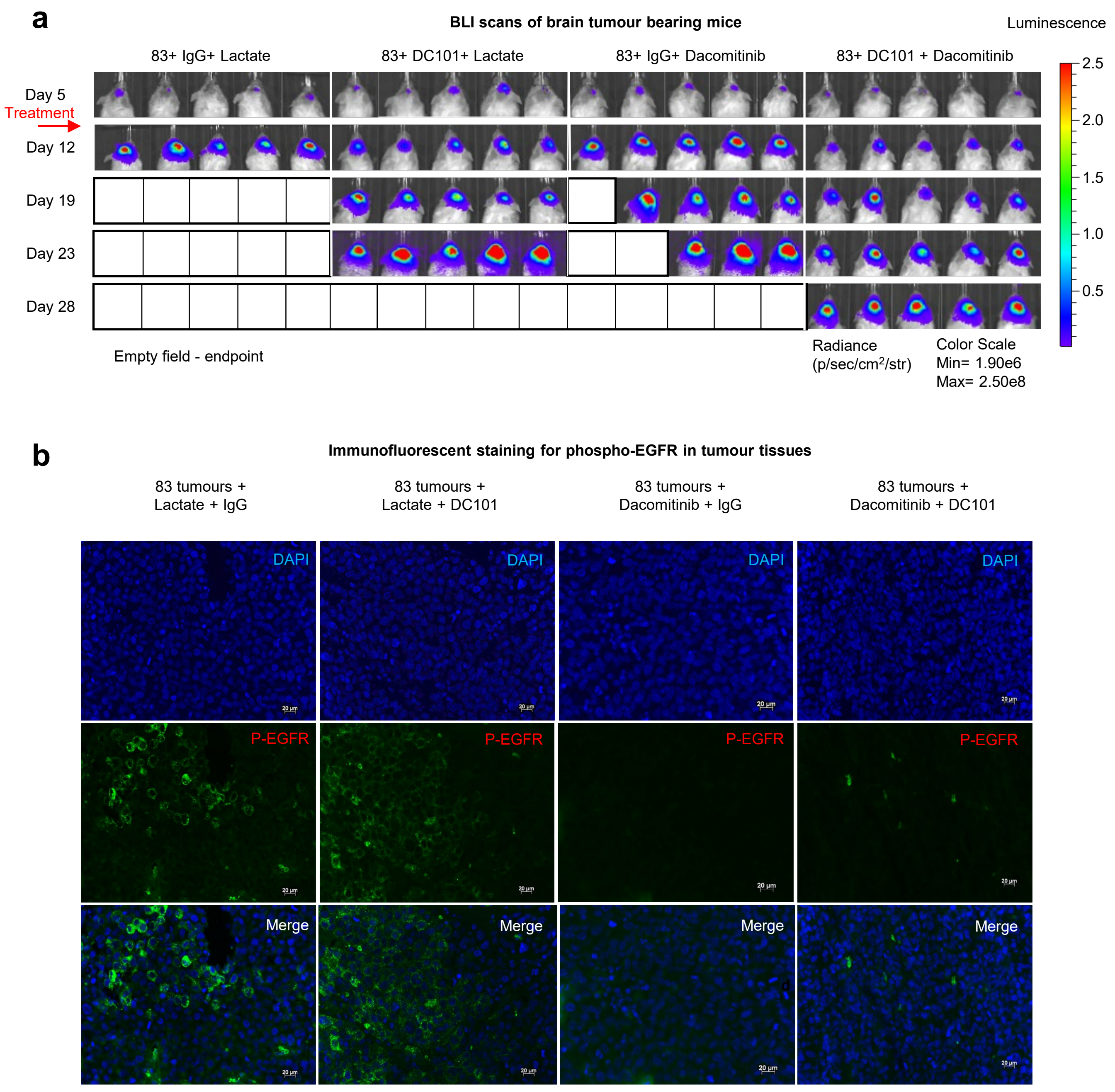

Supplementary Figure 17. Targeting VEGF and EGFR-EV pathways of blood vessel formation in mesenchymal glioma stem cell xenografts. a. Bioluminescent imaging (BLI) of tumours under indicated treatments; **b.** Immunostaining for phospho-EGFR (P-EGFR) in tumour tissues;

Supplementary Materials and Methods

Cell culture conditions

Glioma tumour initiating (stem) cell (GSC) lines were isolated in the laboratory of Dr. Ichiro Nakano (University of Alabama at Birmingham, AL). The two series of cell lines of either proneural (GSC157; GSC1079) or mesenchymal (GSC83; GSC1005) subtype were developed in a form of spheres from surgical samples of glioblastoma (GBM) patients. All GSCs were maintained as spheres in the medium containing DMEM-F12 (GIBCO, Catalog No. 11320033) supplemented with 100μg/ml EGF (GIBCO, Catalog No. PHG0311L), 100μg/ml FGF (GIBCO, Catalog No. PHG0261), 0.2% Heparin (STEMCELL, Catalog No. 07980), 1X B27 serum free supplement (GIBCO, Catalog No. 17504044), 1% Glutamax (GIBCO, Catalog No. 35050061) and 1% penicillin-streptomycin (P/S) (GIBCO, Catalog No. 15070063). EOMA (ATCC, Catalog No. CRL-2586). Mouse hemangioendothelioma (EOMA, transformed endothelial) cells and Human Brain Microvascular Endothelial cells (HBMVEC; iXCells Biotechnologies, Catalog No. 10H-051) were cultured on 0.1% gelatin coated plates. Cells were maintained in DMEM-F12 medium supplemented with 10% FBS, 1% P/S and 40 μg/mL endothelial growth supplement (ECGS) (Sigma, Catalog No. E2759). Immortalized Human Brain Endothelial Cells (HBEC-5i; ATCC, CRL-3245 ™) were cultured in grown in DMEM-F12 media supplemented with 10% FBS, 1% P/S and 40 μg/mL endothelial growth supplement (ECGS; Sigma E2759), as described previously [1]. Human Umbilical Vein Endothelial Cells (HUVEC), normal human primary endothelia, were used from commercially available stocks (ATCC, Catalog No. PCS-100-010) and cultured on 0.1% gelatin coated plates using EGM-2 Bullet-Kit media (Lonza, Catalog No. CC-3162).

Extracellular vesicle isolation

Extracellular vesicles (EVs) were purified by differential centrifugation (Beckman TLA100.2 rotor) from the indicated conditioned media of monolayer cell cultures as described earlier [1]. Briefly, to account for controlled exposure of cells to the fluid phase GSCs were grown as monolayers in their appropriate culture media on laminin (Sigma, L-2020) coated plates and after three days, cell debris was eliminated by centrifugation at 2,000 x g for 20 min. The supernatant was then concentrated (centrifuged at 3,500 x g for 20 min) using Amicon Ultra-15 Centrifugal

Filter Units -100KDa- (Millipore # UFC905008) to a final volume of 1 mL. Concentrated conditioned medium was passed through 0.22μm filters and then centrifuged at 110,000 x g for 70 min. The resulting EV pellet was re-suspended in filtered 1 x PBS or RIPA buffer and stored at -80°C until further use.

RNA extraction and RT-PCR and gel electrophoresis

Total RNA was extracted from cells using TRIzol Reagent (Invitrogen # 15596026) and RNeasy Mini Kit (Qiagen # 74104, Mississauga, ON, Canada) according to manufacturer's recommendations. RNA isolates (50ng) from each cell lines (at least 100,000 cells) were retro-transcribed using QuantiTect Rev. Transcription Kit (Qiagen # 205311). The cDNA obtained was then amplified using Human EGFR primers (EGFR Forward (For): 5'-TGGAGCCTCTTACACCCAG-3' and EGFR Reverse (Rev): 5'-GCTTTCGGAGATGTTGCTTC-3') and EGFRVIII primers (EGFRVIII Forward (For): 5'-CTTCGGGGAGCAGCGATGCGAC-3' and EGFRVIII Reverse (Rev): 5'-ACCAATACCTATTCCGTTACAC -3'). PCR was performed using 2.5 μL of prepared cDNA using MyTaq Red DNA Polymerase (Bioline, London, UK), while 10 μM of EGFR exon 18 primers were used for amplification. PCR was performed for 30 to 33 cycles and amplified PCR products were resolved on 2% agarose gel for 30 minutes at 100V upon which the DNA bands were visualized using ultraviolet (UV) transilluminator gel documentation system.

Nanoparticle tracking analysis

EV size and quantity were analyzed using NS500 (Nanosight; Malvern Panalytical, Malvern, UK) nanoparticle tracking (NTA) instrument. NanoSight technology uses light scattering to visualize particles in the range of 100 nm to 2 μm, records their movement in 30 second video files, tracks individual particles and calculates concentration and size based on Brownian motions. NTA was performed on crude culture media and purified vesicles, as indicated. For culture media, 500 μL aliquot was collected and centrifuged at 400g for 10m to remove cells, then the sample was diluted with D-PBS to reach optimal loading concentration of 10^7 to 10^9 particles per mL. For extracted EVs, the vesicles were diluted 1:200 in D-PBS. Three recordings of 30 seconds were taken for each sample and the analysis was performed as described earlier[2].

Protein quantification and western blot

Total proteins from cells were extracted using RIPA buffer containing 7x protease inhibitor (Roche, Catalog No. 11836153001). Cell lysates were incubated on ice for 5 min, centrifuged at 13,000 rpm for 10 min at 4°C, and solubilized proteins were quantified using the Pierce Micro BCA™ Protein Assay (Thermo Scientific, Rockford, IL, USA). For western blotting (WB) proteins were resolved using sodium dodecyl sulfate–polyacrylamide gel electrophoresis (SDS-PAGE), at 10% gel concentration, and transferred to polyvinylidene difluoride membranes (PVDF; Biorad, Mississauga, ON, Canada). The membranes were probed with indicated primary antibodies, and appropriate horseradish peroxidise (HRP)-conjugated secondary anti-mouse (Biorad # 170-6516, 1:5000), or anti-rabbit (Cell Signaling # 7074S, 1:2000) antibodies. Amersham ECL Western Blotting Detection Kit (RPN2108 GE Healthcare) was used for Detection of Chemiluminescence and band visualization using ChemiDoc MP system (Biorad). Primary antibodies included: rabbit anti-EGFR (4267 Cell signaling, 1:1000), mouse anti-EGFRvIII (bAb0164 Absolute antibody, 1:1000), mouse anti-hEGFR (MA5-13319 Thermo Fisher Scientific, 1:100), rabbit anti-pEGFR Tyr1045 (CS2237 Cell Signalling, 1:1000), rabbit anti-pEGFR Tyr992 (CS2235 Cell Signalling, 1:1000), rabbit anti-pEGFR Tyr1068 (CS2234 Cell Signalling, 1:1000), mouse anti-a/ß actin (A1978 Sigma, 1:3000) and rabbit anti-GAPDH (ABS16 Millipore, 1:1000).

Cell growth/survival assays (MTS Assay)

Cell titer 96 (Promega # 43580) kit was used to measure in vitro cell growth/viability in the presence of Dacomitinib or EGFR knockout. As indicated, 7 x10^3 GSC cells/well were seeded in 96 well plates in full growth media for 24h. The following day the cells were washed and treated with 2.5µM Dacomitinib in DMEM containing 1%FBS. For EGFR-KO and EGFR-WT analysis the cells were left in full growth media for the duration of the assay. The absorbance at 490 nm was read at time intervals indicated, and the signal reflective of viable cell numbers was assessed for up to six days following manufacturer's instruction.

Transwell migration assay

Gelatin (0.1%) coated 8.0µm trans-well inserts (Thermo Fisher Scientific) were placed in 24-well plate and HUVEC (2 x 10^3) cells were plated into the inserts on day 0 of the experiment with full

media. The following day, media was removed, HUVEC cells were washed in PBS twice and starvation media containing 1% EV-depleted FBS[3] was added to the cells and the lower part of the well. Conditioned media (1:1), EV-depleted supernatant (1:1), or reconstituted EVs (30ug/mL), from each cell line, versus control buffer (PBS) containing no EVs were added onto the HUVEC cells to stimulate their migration. After the incubation of 3 days, inserts containing cells were fixed with 3.7% formaldehyde for 5 min and washed twice with PBS. This step was followed by staining with 0.5% crystal violet solution (0.5g crystal violet, 20ml methanol, 80ml water; mixed and filtered through a 0.45µm filter) for 10 min and washed with PBS until no excess stain was left on the inserts. Finally, the non-migrated cells (inside the inserts) were removed manually by gently swabbing the inside surface of each insert using cotton swabs without damaging the insert membrane. Finally, the inserts were examined under the light microscope to evaluate the number of cells that have migrated through the membrane to the bottom surface of the insert. Quantification was performed using FIJI software.

Immunofluorescent staining (IF)

Tumor tissues were preserved in 4% PFA immediately after resection from the mice. They were then run through a series of automated processing steps executed in a Leica TP 1050 tissue processor according to user manual. The resulting paraffin embedded blocks where sectioned using American Optical microtome into 4 µm thick tissue sections, which were placed on permanent positive charge microscope slides and stored until used. Tumor tissue containing slides where de-waxed in Xylene, and then re-hydrated in a series of alcohol washes (95% ethanol - >50%ethanol). Slides were then re-hydrated and antigen retrieval was performed in 0.01 M citrate buffer (pH 6.0) for 20 min. Blocking for 45 min was then performed with PBS containing 5% goat or donkey serum, based on the species of the secondary antibody used. Slides were stained with the following primary antibodies, as indicated: rabbit anti-EGFR (4267 Cell signaling, 1:100), goat anti-mouse CD31(AF3628 R&D Systems, 1:50), rabbit anti-alpha SMA (5694 Abcam, 1:200), rabbit anti-Apelin (702069 Termofisher, 1:50), rabbit anti-VEGFR2 (2479 Cell Signalling, 1:2000), rabbit anti-Survivin (ab182132 abcam, 1:500), rabbit anti-MKi67 (ab264429 abcam, 1:500), rabbit anti-socs2 (MA5-35776 Invitrogen, 1:100), rabbit anti-srsf2 (20371-1-AP Proteintech, 1:200), rabbit anti-pEGFR (2235 Cell signaling, 1:100). As secondary conjugated antibody we used goat anti-Rabbit IgG Alexa Fluor 488 (A-11034 Invitrogen, 1:200) or donkey anti-Goat IgG Alexa Fluor

Plus 594 (A32758 Invitrogen, 1:200). Mounting solution VectaShield® HardSet™ with DAPI was used to seal the slides with coverslip.

Lentivirus production

Lentiviral vectors were used to efficiently introduced genetic or targeting sequences into the cells under investigation. Viral particles were collected from 293T cells subjected to appropriate transfections according to standard protocols and with strict observance of biosafety measures. The day before transfection, $4.5x10^6$ 293T cells were seeded onto 10 cm dish (9mL). On the day of transfection, the plasmid mixture: VSV-G (8454 Addgene), pRRE (12251 Addgene), REV (12253 Addgene) and transfer plasmids were added. As transfer plasmids we used sgRNAs in pCLIP-Dual-SFFV-ZsGreen for EGFR CRISPR guides (TEDH-1024003, TEDH-1024000, TEDH-1024001, TEDH-1055978 Transomic) and pCLIP-Cas9-Nuclease-hCMV-tRFP (SHB_2264 Transomic) for CAS9. Guide RNA plasmid and CAS9 plasmid were obtained from Dr. Sidong Huang, McGill University. For generating luciferase (Luc) positive cells, we used the previously described pSMAL vector modified from the MA1 lentiviral vector to have a Gateway cassette and SFFV promoter (PMID: 15619618 and PMID: 24776803) and with luciferase gene cloned from pGL4.51(luc2/CMV/Neo; E1320 Promega) and kindly provided by Dr. K. Eppert (McGill University). We used 2x HBS mixed with a solution containing 2M Calcium and the plasmid mixture mentioned above for transfection procedure. Transfected cells were then incubated over-night (16-18hr) at 37C, 5% CO2 and on the next day the conditioned media was collected, and debris was spun down at 1,500 x g for 10 min (4°C), Finally, the pellets of viral particles were collected at 22,000 rpm for 2 hrs. These pellets were then re-suspended in 50 µL of PBS. pSMAL lentiviral vectors were used to transduce MES-GSC throughout our experiments. After 16 h, the cells were fed with additional appropriate growth media, and transduction efficiency was measured using flow cytometry for GFP or RFP, as appropriate.

Intracranial injections

The NSG (NOD scid IL2Rgamma-/-) transgenic mice were injected intracranially with GSCs (25,000 cells/µL with total volume of 2µl) using a Stoelting Stereotaxic Injector at pre-determined coordinates (2.5-1.5-3.0) of bregma and sagittal suture, as described [4]. All procedures involving animals were performed in accordance with the guidelines of the Canadian Council of Animal Care (CCAC) and the Animal Utilization Protocols (AUP) approved by the Institutional Animal

Care Committee (ACC) at MUHC RI and McGill University (Protocol #5200). When possible, bioluminescence data was obtained using Xenogen (IVIS 200) bioluminescence scanner, and this was carried out after administering D-Luciferin Firefly potassium salt (Caliper Life Science; 15μg/ml) substrate for 20-30 minutes. These measurements were restricted to mice that had been injected with Luciferase expressing cells and used to visualise the extend and dynamics of tumour formation. Frequent monitoring and humane clinical endpoints were strictly observed.

BME plug vascular growth assay

Cold liquid growth factor-reduced Cultrex basement membrane extract (BME) solution (3433-010-R1 R&D system) was mixed with 100 μg of appropriate GSC EVs or VEGF at indicated concentrations, injected subcutaneously into C57BL/6 mice and allowed to solidify to form a palpable pellet. The injections were all placed at the same location, using a major mammary vessel running along the flank of each mouse and visible through the shaved skin. From the last rib we followed the vessel until its first branching point, which we labeled with a marker and used as the injection point. Pellets were allowed to become vascularized and were collected on day 21 post injection, photographed, imaged under light microscope and placed in sucrose for cryopreservation and histology.

Aortic ring endothelial outgrowth assay

Aortas of 4-week-old C57BL/6 mice were isolated, cleaned of the surrounding tissue and cut into 1 mm long segments (rings). Next, the rings were cultured in growth factor-reduced Cultrex BME (3433-010-R1 R&D system) polymerized at 37 °C. The rings were observed every-day until sprout-like vascular protrusions started to emerge. At that point rings were washed 4 times with PBS and placed in starvation media containing 1% FBS and supplemented with either 30μg/mL of EVs, VEGF, or vehicle. Vascular patterns (angiogenesis and other forms of vascular growth) were assessed at 7 days by using an inverted microscope platform. The number of sprout-like endothelial outgrowths were quantified using Images, which were analyzed using the Fiji distribution of ImageJ (PMID: 22743772) with Angiogenesis Analyzer plugin.

VEGF Elisa

For the detection of VEGF secreted from GSC cells, either as a soluble factor in the supernatant or released in EVs we employed ELISA kit purchased from R&D Systems (#RRV00) and used according to manufacturer's protocol. In order to account for both surface and luminal VEGF, EVs were lysed in RIPA buffer and RIPA was included in, and subtracted from, the assay readings. The readings were collected from three independent samples for each experimental condition, normalized to the total volume and read at 2 times dilutions against a standard curve.

Phosphorylated Receptor Tyrosine Kinase Antibody Array

To demonstrate the intracellular signaling mechanisms elicited by EVs in HUVEC or HBEC-5i cells, cultured endothelial cells were stimulated with indicated EVs at 30 µg/mL. For this purpose GSC- derived EVs were combined with endothelial cells and incubated for 6 days. On the last day, the cells were collected, lysed in RIPA buffer and the relative expression of phosphorylated kinases were analysed using commercial anti-phospho-protein antibody arrays covering 49 different receptor tyrosine kinases (RTKs) and 26 downstream kinases, including 9 mitogen activated protein kinases (MAPKs). The respective kits included Human Phospho-RTK Array kit and the Human Phospho-MAPK Array kit (both from R&D Systems, Minneapolis, MN, USA), respectively, used according to the manufacturer's protocols. Each array was incubated with 250 µg of protein lysate and the levels of phosphorylation were detected by chemiluminescence and quantified using Fiji software. Pixel densities of duplicate spots were averaged, and the value of background was subtracted.

Immunoprecipitation of EGFR-positive EVs

For immunoprecipitation we used Dynabeads™ Protein G, which are uniform, 2.8 µm in diameter, superparamagnetic beads with recombinant Protein G (~17 kDa) covalently coupled to their surfaces. After washing with PBS twice the beads were incubated overnight with rabbit anti-EGFR antibody (4267 Cell signaling). The day after the antibody was removed, beads were washed three times in PBS and incubated overnight with intact EVs (30ug). Using a magnet, EVs enriched with EGFR proteins were separated from the flow through fraction, which contained EVs not carrying EGFR proteins. The EGFR-positive EVs and EGFR-negative EVs were then lysed either in RIPA buffer or in Lysis buffer for RNA extraction (as mentioned above). Protein validation was

performed by western blotting to ensure complete separation of EGFR antigen-positive and EGFR antigen-negative EVs, and EGFR mRNA was tested by RT-PCR.

CRISPR-CAS 9 gene editing

EGFR knock out in GSC was performed by means of lentiviral transduction, with the vector described above. Target GSC cells were first transduced with CAS9-RFP containing virus. Positive cells were then sorted by fluorescence activated cell sorting (FACS), after which RFP positive cells were transduced with three different viruses (each of them carrying two guide RNAs and GFP cassette). After 4 washes in PBS the transduced cells were stained with APC-conjugated anti-human EGFR antibody (352906 Biolegend, 1:20). Single clones that were positive for RFP and GFP, but negative for APC were then sorted into different wells of a 96 well plate. EGFR-negative cells were then validated by EGFR western blotting and targeted *EGFR* gene sequencing, which revealed disruption of the entire *EGFR* sequence, thereby preventing the expression of both wild type (EGFR) and variant III (EGFRvIII) isoforms.

EGFR rescue experiments

In order to validate the EGFR-specificity of biological effects observed upon *EGFR* gene editing we selectively reconstituted EGFRvIII expression in these cells and tested their responses and EV properties. This was accomplished by stable transfections of EGFRvIII, which were carried out in MES GSCs using pLNCX modified plasmid as described previously[5]. This plasmid contained human *EGFRvIII* sequence to ensure a selective re-expression of this oncogene in previously edited cells. An empty vector was used for control (mock) transfections. Cells were grown on 6 well (FALCON) plates to 70% confluency, at which point they were starved in serum free (0% FBS) media overnight. The cultures were then exposed to 1 μg/ul of pLKO.1 sterile vector DNA mixed with 5 μl OPTI-DMEM (GIBCO) and 5 μL of Lipofectamin 2000 (Invitrogen) and incubated for additional 6 hours. The media was then replaced with GSC culture media (DMEM-F12) prior to testing for EGFRvIII expression. To accomplish this the media were removed and after 4 washes with PBS the cells were stained with APC-conjugated anti-human EGFR antibody which detects both isoforms of EGFR (352906 Biolegend, 1:20). Single clones that were positive for EGFR-APC were then sorted in different wells of a 96 well plate. EGFR-positive cells were then validated by western blotting. This procedure selectively reconstituted EGFRvIII expression

in *EGFR*-KO cells and thereby enabled us to attribute the biological effects observed to this oncogene.

GeoMx™ DSP with mouse WTA

GeoMx™ digital spatial profiling (DSP) was performed as per the supplier's protocol using the mouse Whole Transcriptome Atlas (WTA) probe set with read-out baaed on next-generation sequencing (Nanostring Technologies). Briefly, 5 µm FFPE sections of tumour bearing mouse brains injected with either EGFR-KO or EGFR-WT glioblastoma cells, or control mouse brains, were processed through deparaffinization, antigen retrieval (in Tris-EDTA pH9 for 20 min at 98°C), and Proteinase K digestion (0.1 ug/mL Proteinase K for 15 min at 37°C) prior to overnight incubation at 37°C with the mouse Whole Transcriptome Atlas diluted in hybridization Buffer R. The next day, slides were washed twice in 2XSSC/formamide (25 min at 37°C), blocked with Buffer W (30 min at RT) and incubated with anti-CD31 primary antibody (AF3628 R&D Systems, diluted 1:20 in Buffer W) for 1 hr in a humidity chamber. Slides were washed 3 times in 2XSSC and incubated with anti-mouse- Alexa Fluor Plus 594 antibody (A32758 Invitrogen, diluted 1:2000 in Buffer W) plus 500 nM SYTO13 for 1 hr at room temperature (RT). Following 2 washes with 2XSSC, slides were scanned on the GeoMx™ DSP instrument (at 20X) and Whole Transcriptome barcodes from areas containing CD31-positive blood vessels were released from pre-defined regions-of-interest (ROI; labelled with anti-CD31 antibody) by UV light illumination and microfluidic collection. The UV-released barcodes from each area-of-illumination (AOI) were PCR-amplified to add unique dual-index Illumina i5 and i7 indices and then bead-purified twice using AMPure XP beads (Beckman Coulter, A63881). Pooled libraries were sequenced on Illumina NovaSeq platform, generating 68M reads. FASTQ files were processed by NanoString's GeoMx NGS Pipeline (DND) and uploaded into the GeoMx system for data analysis. Outlier WTA probes were removed prior to generating a consensus count for each target; outliers were identified as any probe with counts 90% below the mean of the probe group in at least 20% of the AOIs analyzed (using the Grubbs test). Targets were then filtered out if expressed at less than the limit of quantitation (LOQ) in at least 5% of the AOIs. The counts for each target transcript in each AOI were then normalized to the counts of the top 25% expressed genes in the respective AOI (Q3 normalization). Differential gene expression analysis was performed on the GeoMx DA software

using Linear Mixed Model with Benjamini-Hochberg correction statistical tests for pair-wise comparisons.

Lycopersicon lectin injection and vibratome sectioning to visualize perfused blood vessels

Intracranial injections were prepared by injecting 25 x 10^3 GSCs into the brains of NSC mice (Charles River Labs). For staining blood vessels, Lycopersicon lectin (DL-1178, vector Laboratories) was injected i.v. and allowed to circulate and bind to the endothelium for 30 min prior to humane euthanasia. Brains from mice were harvested and immediately immersed in cold PBS. The brains were then sectioned at 200 μm using vibratome (Leica VT 1200s). The tissue sections were placed in a μ-Dish 35 mm, high Glass Bottom dish (81158, ibidi) and taken for high resolution confocal microscopy (Zeiss LSM780 laser scanning confocal microscope).

Flow Cytometry

EOMA endothelial cells were treated with EVs derived from GSC157 for 24h, then stained with anti-CD31 antibody (AF3628 R&D Systems), mouse IgG1 (400112, Biolegend), human specific anti-CD133 antibody (372805, Biolegend) or CellTrace™ Far Red (C34572 Thermo Fisher). The cells were subjected to flow cytometry (BD LSR Fortessa) and data was analyzed using FlowJo software. Mouse endothelial cells were isolated 15 days after GSC83 injections, stained for CD31 (AF3628 R&D Systems, 1:50), EGFR (352904, biolegend, 1:20), or permeabilized and stained for pEGFR Tyr1045 (CS2237 Cell Signalling, 1:50). The intact (non-permeabilized) cells were sorted using BD FACSAria Fusion 1, while the other cells were subjected to beads-based isolation using autoMACS Pro Separator (Miltenyi Biotec).

Single cell mRNA sequencing, data processing and analysis

Whole-tumor cells were dissociated and isolated using Collagenase/Dispase (11097113001 Millipore) and resuspended in PBS for single-cell capture. Following sample preparation according to Chromium Single Cell 3' Reagent Kits v3 User Guide (CG0052 10x Genomics)[6] , a single-cell RNA library was generated using the GemCode Single-Cell Instrument (10x Genomics, Pleasanton, CA, USA). The sequencing ready library was purified with SPRIselect, quality

controlled for size distribution and yield (LabChip GX Perkin Elmer) and quantified using qPCR (KAPA Biosystems Library Quantification Kit for Illumina platforms P/N KK4824). We used Cell Ranger v3.0.1 (10x Genomics) to demultiplex the raw sequencing reads to FASTQ files and align the reads to human and mouse reference hg19 and mm10 to quantify gene counts for each origin of species as per unique molecular identifiers (UMIs), getting about 349 million and 404 million read counts for EGFR-WT and -KO samples, respectively. We loaded the gene count data using the Seurat package and separated the object to human and mouse data for further quality control. Due to the overall differences in normal distribution of the number of UMIs and the percentage of mitochondrial genes between the two species, we applied criteria differently for each species. For human cells, we excluded cells with less than 5,000 UMIs and over 15% of mitochondrial genes in the total human gene counts. After filtering, we removed murine gene counts from the human object for further analysis pipeline, excluding cells with less than 500 murine UMIs and over 10% mitochondrial genes in the total murine gene counts. Doublets were identified using lineage-specific marker sets identified in clustering analysis. We considered cells expressing two or more sets of murine cell lineage-specific marker genes as intra-species doublets. We also excluded inter-species doublets to avoid expression of human EGFR from inter-species doublets. Human genes were removed from the murine object for further analysis such as normalization and dimension reduction steps but retrieved only for data visualization. Each object was processed basically based on the Seurat pipeline, and the normalized data was visualized using the Seurat and dittoSeq packages.

RNAscope® fluorescent multiplex gene expression analysis

Four mouse brain FFPE samples - Two (2) WT and Two (2) KO samples were fixed in 4% PFA overnight at room temperature and embedded in paraffin blocks. FFPE blocks were cut to obtain 5 μm thick sections which were mounted onto Superfrost Plus slides. The following probes were customized by ACDBio: Mm-Top2a-No-XHs-C1 (Catalog No. 1235878-C1), Mm-Pecam1-No-XHs-C2 (Catalog No. 451508-C2), Mm-Birc5-No-XHs-C3 (Catalog No. 1235898-C3), Mm-Socs2-No-XHs-C4 (Catalog No. 1235928-C4), Mm- Mm-Fbl-No-XHs-C1 (Catalog No. 1245068-C1), Mm-Mt2-No-XHs-C3 (Catalog No. 1245078-C3), Mm-Srsf2-No-XHs-C4 (Catalog No. 1235918-C4). RNA QC and staining were performed by the company according to manufacturer's protocol.

Data collection and statistical analysis

All experiments were reproduced at least 2-3 times with similar results, unless otherwise indicated. Statistical analysis was carried out using a computerized two-tailed Student's t-test and ANOVA. Error bars represent standard deviation. * indicates significance <0.05, ** indicates significance <0.01, *** indicates significance <0.001 and **** indicates significance <0.0001.

Supplementary Methods - References

1 Spinelli, C. *et al.* Molecular subtypes and differentiation programmes of glioma stem cells as determinants of extracellular vesicle profiles and endothelial cell-stimulating activities. *J Extracell Vesicles* **7**, 1490144 (2018). https://doi.org:10.1080/20013078.2018.1490144

2 Montermini, L. *et al.* Inhibition of oncogenic epidermal growth factor receptor kinase triggers release of exosome-like extracellular vesicles and impacts their phosphoprotein and DNA content. *J Biol Chem* **290**, 24534-24546 (2015). https://doi.org:10.1074/jbc.M115.679217

3 Choi, D. *et al.* Oncogenic RAS drives the CRAF-dependent extracellular vesicle uptake mechanism coupled with metastasis. *J Extracell Vesicles* **10**, e12091 (2021). https://doi.org:10.1002/jev2.12091

4 Garnier, D. *et al.* Divergent evolution of temozolomide resistance in glioblastoma stem cells is reflected in extracellular vesicles and coupled with radiosensitization. *Neuro Oncol* **20**, 236-248 (2018). https://doi.org:10.1093/neuonc/nox142

5 Al-Nedawi, K. *et al.* Intercellular transfer of the oncogenic receptor EGFRvIII by microvesicles derived from tumour cells. *Nat Cell Biol* **10**, 619-624 (2008). https://doi.org:10.1038/ncb1725

6 Zheng, G. X. *et al.* Massively parallel digital transcriptional profiling of single cells. *Nat Commun* **8**, 14049 (2017). https://doi.org:10.1038/ncomms14049
