# Supplementary material for: Mesenchymal glioma stem cells trigger vasectasia—distinct neovascularization process stimulated by extracellular vesicles carrying EGFR
Source: Nat Commun. 2024 Apr 3;15:2865. doi: 10.1038/s41467-024-46597-x (PMC10991552; doi:10.1038/s41467-024-46597-x)
Supplement: Supplementary file 3 — Reporting Summary [file 41467_2024_46597_MOESM3_ESM.pdf]

nature portfolio

Corresponding author(s): Janusz Rak

Last updated by author(s): Feb 16, 2024

Reporting Summary

Nature Portfolio wishes to improve the reproducibility of the work that we publish. This form provides structure for consistency and transparency in reporting. For further information on Nature Portfolio policies, see our Editorial Policies and the Editorial Policy Checklist.

Statistics

For all statistical analyses, confirm that the following items are present in the figure legend, table legend, main text, or Methods section.

n/a	Confirmed	
☐	☒	The exact sample size (*n*) for each experimental group/condition, given as a discrete number and unit of measurement
☐	☒	A statement on whether measurements were taken from distinct samples or whether the same sample was measured repeatedly
☐	☒	The statistical test(s) used AND whether they are one- or two-sided<br>*Only common tests should be described solely by name; describe more complex techniques in the Methods section.*
☒	☐	A description of all covariates tested
☐	☒	A description of any assumptions or corrections, such as tests of normality and adjustment for multiple comparisons
☐	☒	A full description of the statistical parameters including central tendency (e.g. means) or other basic estimates (e.g. regression coefficient) AND variation (e.g. standard deviation) or associated estimates of uncertainty (e.g. confidence intervals)
☐	☒	For null hypothesis testing, the test statistic (e.g. *F*, *t*, *r*) with confidence intervals, effect sizes, degrees of freedom and *P* value noted<br>*Give P values as exact values whenever suitable.*
☒	☐	For Bayesian analysis, information on the choice of priors and Markov chain Monte Carlo settings
☒	☐	For hierarchical and complex designs, identification of the appropriate level for tests and full reporting of outcomes
☒	☐	Estimates of effect sizes (e.g. Cohen's *d*, Pearson's *r*), indicating how they were calculated

Our web collection on statistics for biologists contains articles on many of the points above.

Software and code

Policy information about availability of computer code

Data collection	Cell Ranger v3.0.1<br>GeoMx DA software
Data analysis	Prism 9, ImageJ2(FIJI), ImageLab 6.0, TBtools, GSEA_4.1.0, Scaffold4, GeoMx system. For single cell sequencing we used Cell Ranger v3.0.1 (10x Genomics) to demultiplex the raw sequencing reads to FASTQ files and used Seurat package (Satija, R. t al.). Statistical analysis and visualization of gene sets were performed using the clusterProfiler R package (Yu, G., Wang L.G. et al).

For manuscripts utilizing custom algorithms or software that are central to the research but not yet described in published literature, software must be made available to editors and reviewers. We strongly encourage code deposition in a community repository (e.g. GitHub). See the Nature Portfolio guidelines for submitting code & software for further information.

Data

Policy information about availability of data

All manuscripts must include a data availability statement. This statement should provide the following information, where applicable:

- Accession codes, unique identifiers, or web links for publicly available datasets
- A description of any restrictions on data availability
- For clinical datasets or third party data, please ensure that the statement adheres to our policy

The single cell sequencing data generated in this study have been deposited as unfiltered and filtered R objects as well as raw data in the Gene Expression Omnibus

database under accession code GSE207360 [https://www.ncbi.nlm.nih.gov/geo/query/acc.cgi?acc=GSE207360]. For the whole transcriptome digital spatial profiling data, the data are available as a supplement to the manuscript in the formats of raw expression (DCC and PKC files) and processed expression (Q3-normalized and with genes detected in over 5% of the samples) data along with the annotation file for each ROI. Source data for main and Supplementary figures are provided as a separate file along with corresponding Source Data file. All other information is available on request. Code availability The code is available on the Seurat (https://satijalab.org/seurat/) and GeoScript Hub (https://nanostring.com/products/geomx-digital-spatial-profiler/geoscript-hub/). The scripts used for the figures are available on request.

Research involving human participants, their data, or biological material

Policy information about studies with human participants or human data. See also policy information about sex, gender (identity/presentation), and sexual orientation and race, ethnicity and racism.

Reporting on sex and gender	N/A
Reporting on race, ethnicity, or other socially relevant groupings	N/A
Population characteristics	N/A
Recruitment	N/A
Ethics oversight	N/A

Note that full information on the approval of the study protocol must also be provided in the manuscript.

Field-specific reporting

Please select the one below that is the best fit for your research. If you are not sure, read the appropriate sections before making your selection.

☒ Life sciences ☐ Behavioural & social sciences ☐ Ecological, evolutionary & environmental sciences

For a reference copy of the document with all sections, see nature.com/documents/nr-reporting-summary-flat.pdf

Life sciences study design

All studies must disclose on these points even when the disclosure is negative.

Sample size	The sample size was equal or larger than 3 for most experiments, while for intra-cranial injections and tumour staining the sample size was equal or larger than 5. The sample size of five mice per condition was determined considering resource constraints and the resulting statistical significance underscores the observed effects in the study. 50-50% distribution between male and female mice and simple random method was used for data sampling. We applied standard statistical methods and all data referred to as significant has p<0.05 for all the experimental quantification and Analytical/Molecular techniques.
Data exclusions	Data was no excluded from experiments.
Replication	We replicated and repeated all our experiments a minimum of three times in order to allow statistical quantification.
Randomization	All mice/ experiments were randomly assigned to treated or no-treated.
Blinding	The investigators were not blinded to the design of the study due to the experimental conditions. However during injections of tumor cells the mouse surgeon was blinded to the experimental condition. Mice were grouped according to the vial of cells injected which were labeled with a specific code blinded to the observer. All images were taken as per the group. For sex distribution, mice were randomly taken at surgery to have equal numbers of mice for each sex. Mice were housed individually post surgery.

Reporting for specific materials, systems and methods

We require information from authors about some types of materials, experimental systems and methods used in many studies. Here, indicate whether each material, system or method listed is relevant to your study. If you are not sure if a list item applies to your research, read the appropriate section before selecting a response.

Materials & experimental systems

n/a	Involved in the study
☐	☒ Antibodies
☐	☒ Eukaryotic cell lines
☒	☐ Palaeontology and archaeology
☐	☒ Animals and other organisms
☒	☐ Clinical data
☒	☐ Dual use research of concern
☒	☐ Plants

Methods

n/a	Involved in the study
☒	☐ ChIP-seq
☒	☐ Flow cytometry
☒	☐ MRI-based neuroimaging

Antibodies

Antibodies used

Primary antibodies:
Rabbit anti-EGFR (4267 Cell signaling, Clone D38B1, 1:1000 or 1:100)
Mouse anti-EGFRvIII (bAb0164 Absolute antibody, Clone L8A4, 1:1000)
Mouse anti-hEGFR (MA5-13319 Thermo Fisher Scientific, Clone 199.12, 1:100)
Rabbit anti-pEGFR Tyr1045 (CS2237 Cell Signalling, polyclonal, 1:1000 or 1:50)
Rabbit anti-pEGFR Tyr992 (CS2235 Cell Signalling, polyclonal, 1:1000 or 1:100)
Rabbit anti-pEGFR Tyr1068 (CS2234 Cell Signalling, Clone D7A5, 1:1000)
Goat anti-mouse CD31(AF3628 R&D Systems, polyclonal, 1:50)
Rabbit anti-alpha SMA (5694 Abcam, polyclonal, 1:200)
APC-conjugated anti-human EGFR antibody (352906 Biolegend, Clone AY13, 1:20)
PE-conjugated anti-human EGFR antibody (352904 Biolegend, Clone AY13, 1:20)
Mouse anti-IgG1 (400112 Biolegend, Clone MOPC-21, 1:40)
APC anti-human CD133 Antibody (372805 Biolegend, Clone 7, 1:20)
Mouse anti-a/ß Actin (A1978 Sigma, Clone AC-15, 1:3000)
Rabbit anti-GAPDH (ABS16 Millipore, polyclonal, 1:1000)
Rabbit anti-Apelin (702069 Termofisher, Clone 5H5L9, 1:50)
Rabbit anti-VEGFR2 (2479 Cell Signalling, Clone 55B11, 1:2000)
Rabbit anti-Survivin (ab182132 abcam, Clone EPR17358, 1:500 or 1:1000)
Rabbit anti-MKi67 (ab264429 abcam, polyclonal, 1:500)
Rabbit anti-socs2 (MA5-35776 Invitrogen, Clone ARC1470, 1:100)
Rabbit anti-srsf2 (20371-1-AP Proteintech, polyclonal, 1:200)

Secondary antibodies:
HRP-anti rabbit (7074 Cell Signalling, polyclonal, 1:2000)
HRP-anti mouse (170-6516 Biorad, polyclonal. 1:5000)
Goat anti-Rabbit IgG Alexa Fluor 488 (A-11034 Invitrogen, polyclonal, 1:200)
Donkey anti-Goat IgG Alexa Fluor Plus 594 (A32758 Invitrogen, polyclonal, 1:200)

Validation

Rabbit anti-EGFR, monoclonal antibody, validated for WB, IP, IHC, IF, Flow Cyt in Human, Mouse and Monkey. https://www.cellsignal.com/products/primary-antibodies/egf-receptor-d38b1-xp-rabbit-mab/4267
Mouse anti-EGFRvIII, monoclonal antibody, validated for WB, ELISA, FC, IF, IHC in Human. https://absoluteantibody.com/product/anti-egfrviii-l8a4/
Mouse anti-hEGFR, monoclonal antibody, validated for WB, IP, IF, IHC in Human. https://www.fishersci.com/shop/products/egfr-monoclonal-antibody-199-12-invitrogen/PIMA513319
Rabbit anti-pEGFR Tyr1045, polyclonal antibody, validated for WB, IF, ICC in Human and Rat. https://www.cellsignal.com/products/primary-antibodies/phospho-egf-receptor-tyr1045-antibody/2237
Rabbit anti-pEGFR Tyr992, polyclonal antibody, validated for WB, IHC in Human, Mouse and Monkey. https://www.cellsignal.com/products/primary-antibodies/phospho-egf-receptor-tyr992-antibody/2235
Rabbit anti-pEGFR Tyr1068, polyclonal antibody, validated for WB, IHC in Human, Mouse and Rat. https://www.cellsignal.com/products/primary-antibodies/phospho-egf-receptor-tyr1068-d7a5-xp-rabbit-mab/3777
Goat anti-mouse CD31, polyclonal antibody, validated for WB, Flow Cyt, ICC, IHC in Mouse and Rat. https://www.rndsystems.com/products/human-mouse-rat-cd31-pecam-1-antibody_af3628#product-datasheets
Rabbit anti-alpha SMA, polyclonal antibody, validated for WB and IHC in Human and Mouse. https://www.abcam.com/products/primary-antibodies/alpha-smooth-muscle-actin-antibody-ab5694.html
APC-conjugated anti-human EGFR, monoclonal antibody, validated for Flow Cyt in Mouse. https://www.biolegend.com/en-gb/products/apc-anti-human-egfr-antibody-7714?GroupID=BLG9533
PE-conjugated anti-human EGFR, monoclonal antibody, validated for Flow Cyt in Mouse. https://www.biolegend.com/fr-fr/productstab/pe-anti-human-egfr-antibody-7432
Mouse anti-IgG1 monoclonal antibody, validated for Flow Cyt in Human, Mouse and Rat. https://www.biolegend.com/nl-nl/products/pe-mouse-igg1-kappa-isotype-ctrl-1408
APC anti-human CD133 Antibody, monoclonal antibody, validated for Flow Cyt in Human. https://www.biolegend.com/fr-fr/products/apc-anti-human-cd133-antibody-13915?GroupID=BLG15840
Mouse anti-a/ß Actin, monoclonal antibody, validated for WB, IP, IHC, IF in Human, Mouse and Rat. https://www.sigmaaldrich.com/CA/en/product/sigma/a1978
Rabbit anti-GAPDH,polyclonal antibody, validated for Human, Mouse and Rat. https://www.sigmaaldrich.com/CA/en/product/mm/abs16?utm_source=google&utm_medium=cpc&utm_campaign=20674735577&utm_content=155564356500&gclid=CjwKCAiAibeuBhAAEiwAiXBoJIBzOWrU2bV3q9D2UuHBj1QbXGSkuEmcsfdx4UbADA8B0CucAYHOshoCE8gQAvD_BwE

Rabbit anti-Apelin, monoclonal antibody, validated for WB,ICC, IHC, IF in Human and Mouse. https://www.thermofisher.com/antibody/product/Apelin-Receptor-Antibody-clone-5H5L9-Recombinant-Monoclonal/702069
Rabbit anti-VEGFR2, monoclonal antibody, validated for WB, IP, ICC, IHC, IF in Human and Mouse. https://www.cellsignal.com/products/primary-antibodies/vegf-receptor-2-55b11-rabbit-mab/2479
Rabbit anti-Survivin, monoclonal antibody, validated for WB, IP, IHC, in Rat and Mouse. https://www.abcam.com/products/primary-antibodies/survivin-antibody-epr17358-ab182132.html
Rabbit anti-MKi67, polyclonal antibody, validated for IHC in Mouse. https://www.abcam.com/products/primary-antibodies/ki67-antibody-ab264429.html
Rabbit anti-socs2, monoclonal antibody, validated for WB, IHC, IF in Human, Mouse and Rat. https://www.thermofisher.com/antibody/product/SOCS2-Antibody-clone-ARC1470-Recombinant-Monoclonal/MA5-35776
Rabbit anti-srsf2, polyclonal antibody, validated for WB, IP, IHC, IF, CoIP, ELISA, in Human, Mouse and Rat. https://www.ptglab.com/products/SFRS2-Antibody-20371-1-AP.htm
HRP-anti rabbit, designed for use with rabbit polyclonal and monoclonal antibodies,validated for WB. https://www.cellsignal.com/products/secondary-antibodies/anti-rabbit-igg-hrp-linked-antibody/7074
HRP-anti mouse, designed for use with mouse polyclonal and monoclonal antibodies,validated for WB. https://www.bio-rad.com/en-ca/sku/1706516-goat-anti-mouse-igg-h-l-hrp-conjugate?ID=1706516
Goat anti-Rabbit IgG Alexa Fluor 488, designed for use with mouse polyclonal and monoclonal antibodies, validated for ICC, IF, IHC and Flow Cyt. https://www.thermofisher.com/antibody/product/Goat-anti-Rabbit-IgG-H-L-Highly-Cross-Adsorbed-Secondary-Antibody-Polyclonal/A-11034
Donkey anti-Goat IgG Alexa Fluor Plus 594, designed for use with goat polyclonal and monoclonal antibodies, validated for ICC, IF, and IHC. https://www.thermofisher.com/antibody/product/Donkey-anti-Goat-IgG-H-L-Highly-Cross-Adsorbed-Secondary-Antibody-Polyclonal/A32758
All antibodies, as indicated at the manufacturers' websites, have multiple citations serving as additional validation.

Eukaryotic cell lines

Policy information about cell lines and Sex and Gender in Research

Cell line source(s)	HUVEC (Catalog No. PCS-100-010), EOMA (Catalog No. CRL-2586) and HBEC-5i cells were obtained from ATCC and cultured in the recommended media or as indicated. MBMVEC cells were purchased from iXCells Biotechnologies (Catalog No. 10HU-051)<br>Patient derived glioma stem cell, both proneural (GSC157; GSC1079) and mesenchymal (GSC83; GSC1005) subtype were isolated from male (GSC1079; GSC1005) or female (GSC157; GSC83) patients with Glioblastoma tumour, as previously described (Mao et al. 2013).<br>Glioma stem cells with stable knockdown of EGFR (83-KO-19, 83-KO-27, 1005-KO-11 and 1005-KO-14) were constructed by lentiviral transduction using the CRISPR-Cas9 system (EGFR CRISPR guides: TEDH-1024003, TEDH-1024000, TEDH-1024001, TEDH-1055978 and Cas9 SHB_2264 from Transomic).<br>Luciferase positive 83 GSC were constructed by lentiviral transduction using the pSMAL vector modified from the MA1 lentiviral vector to have a Gateway cassette and SFFV promoter and with Luciferase gene cloned from pGL4.51(luc2/CMV/Neo from Promega) (kindly obtained from Dr. K. Eppert, McGill University).<br>EGFR rescued cells (83-KO-19 EGFR + and 1005-KO-14 EGFR +) were obtained by EGFR stable transfection using pLNCX modified plasmid as described previously (Al-Nedawi 2008), containing human EGFRvIII. The empty vector was used for control cell line (83-KO-19 pLNCX and 1005-KO-14-pLNCX)
Authentication	Purchased cell lines underwent validation through both commercial vendor assessments and evaluations based on morphology in our laboratory. All human glioma stem cell lines were validated in the original studies (Mao 2013, Chandran 2015) by various means such as RNA seq, staining for pluripotency markers and determining differentiation capacity.<br>All the transduced cell lines in the laboratory were validated by western blot and targeted sequencing.
Mycoplasma contamination	All cells were tested to be free of Mycoplasma contamination.
Commonly misidentified lines<br>(See ICLAC register)	No cell lines used in this study were found in the database of commonly misidentified cell lines that is maintained by ICLAC and NCBI Biosample.

Animals and other research organisms

Policy information about studies involving animals; ARRIVE guidelines recommended for reporting animal research, and Sex and Gender in Research

Laboratory animals	All mice used in this study were obtained from the Jackson Laboratory. NOD scid IL2Rgamma-null (NSG) transgenic mice were chosen between 2 and 6 months of age for intra-cranial injections.<br>C57BL/6 mice were used for BME plug assay and isolation of aortic rings at the age of 4 weeks. All mice were maintained at MUHC RI and McGill University animal care facility, under 12 hours of light/12 hours of dark cycle. Ambient temperature for housing the mice was between 20-26 degrees Celsius (68-79 degrees Fahrenheit), with a humidity level of 30-70%.
Wild animals	This study did not involve wild animals.
Reporting on sex	For all the in vivo studies both animal sex were taken in consideration. We used 50% female and 50% male in both treated and untreated conditions.
Field-collected samples	This study did not involve field-collected samples
Ethics oversight	All procedures involving animals were performed in accordance with the guidelines of the Canadian Council of Animal Care (CCAC)

Ethics oversight	and the Animal Utilization Protocols (AUP) were approved by the Institutional Animal Care Committee (ACC) at MUHC RI and McGill University (Protocol #5200)

Note that full information on the approval of the study protocol must also be provided in the manuscript.

Plants

Seed stocks	N/A
Novel plant genotypes	N/A
Authentication	N/A
